# Graphene-based Membranes for H_2_ Separation: Recent Progress and Future Perspective

**DOI:** 10.3390/membranes10110336

**Published:** 2020-11-12

**Authors:** Chong Yang Chuah, Jaewon Lee, Tae-Hyun Bae

**Affiliations:** 1 Singapore Membrane Technology Centre, Nanyang Environment and Water Research Institute, Nanyang Technological University, Singapore 637141, Singapore; chongyang.chuah@ntu.edu.sg; 2 Department of Chemical and Biomolecular Engineering, Korea Advanced Institute of Science and Technology, Daejeon 34141, Korea; jaewon_lee@kaist.ac.kr

**Keywords:** grapheme, membrane, H_2_ separation, permeability, selectivity, upper bound

## Abstract

Hydrogen (H_2_) is an industrial gas that has showcased its importance in several well-known processes such as ammonia, methanol and steel productions, as well as in petrochemical industries. Besides, there is a growing interest in H_2_ production and purification owing to the global efforts to minimize the emission of greenhouse gases. Nevertheless, H_2_ which is produced synthetically is expected to contain other impurities and unreacted substituents (e.g., carbon dioxide, CO_2_; nitrogen, N_2_ and methane, CH_4_), such that subsequent purification steps are typically required for practical applications. In this context, membrane-based separation has attracted a vast amount of interest due to its desirable advantages over conventional separation processes, such as the ease of operation, low energy consumption and small plant footprint. Efforts have also been made for the development of high-performance membranes that can overcome the limitations of conventional polymer membranes. In particular, the studies on graphene-based membranes have been actively conducted most recently, showcasing outstanding H_2_-separation performances. This review focuses on the recent progress and potential challenges in graphene-based membranes for H_2_ purification.

## 1. Introduction

Hydrogen (H_2_) is an important industrial gas that is heavily utilized in the petrochemical industries. For instance, H_2_ is involved in the hydrodesulfurization process, which removes sulfur from natural gas [1,2] for subsequent petroleum refining process. In addition, H_2_ is also employed as the main reactant in productions of ammonia, methanol and steel [3,4,5]. More importantly, in recent years, H_2_ has attracted a large amount of attentions as a carbon-free energy resource possessing the highest energy density per unit mass (120–142 MJ/kg). This is because the use of H_2_ produces water as the only byproduct [6], in contrast to conventional fossil fuels that inevitably emit carbon dioxide (CO_2_), a greenhouse gas [7,8]. This behavior has led to a rapid increase in demand of hydrogen, such that the reported global demand is registered at 73.9 megatons (Mt) in 2018 [9].

In general, H_2_ is produced by using the natural gas (mainly methane (CH_4_)) via a steam-reforming process, with the aid of high-temperature steam. It should be noted that this process accounts for the major portion in global H_2_ production in comparison to other alternative approaches, such as partial methane oxidation and water splitting [10]. However, this process inevitably generates undesirable impurities, such as CO_2_, CH_4_ and nitrogen (N_2_). Notably, it was reported that the steam-reforming accounts for approximately 830 million tons of CO_2_ emission annually [9]. On the other hand, environmentally-benign process such as water splitting via electrolysis or photolysis is able to satisfy a mere 3.9% of the global demand, such that, at current stage, it is difficult to phase out the conventional process for H_2_ production [11,12]. In this regard, it is necessary to separate H_2_ from other components, particularly CO_2_ and CH_4_, which are classified as greenhouse gases under the Kyoto Protocol [13,14,15,16].

The separation of H_2_ from gas mixtures can be conducted using pressure-swing adsorption (PSA) and cryogenic distillation, allowing the production of high-purity H_2_ [17,18,19,20,21]. However, both processes generally require extensive compression work and large energy penalties, leading to a high production cost [22,23,24]. Therefore, membrane-based separation has been considered as an alternative unit operation due to its simple operation, small plant footprint and high energy efficiency [25,26,27]. In general, polymeric membranes are commonly utilized in gas separations due to an excellent processability. This allows the fabrication of membranes into various forms, including spiral wound or hollow fiber, a well-established and scalable synthesis process together with a desirable mechanical stability. However, the performance of the polymeric membrane is limited by the trade-off relationship between permeability and selectivity, as evidenced by the Robeson plot [28,29]. It is considerably challenging to optimize both permeability and selectivity in polymeric membranes, as the gas transport properties are governed by the solution-diffusion mechanism [30,31,32]. Efforts in improving the gas separation performance have been made by employing molecular sieves such as zeolites, metal-organic frameworks (MOFs) and microporous organic polymers (MOPs) as membrane materials. However, these membranes suffer from limited scalability and poor mechanical stability [33,34].

In recent years, various two-dimensional (2D) materials such as MXene [35,36], transition metal dichalcogenides (e.g., MoS_2_-TMD) [37], layered double hydroxides (NiAl-CO_3_-LDH) [38], MOF-based nanosheets [39], covalent organic framework (COF)-based nanosheets [40], graphene [41] and carbon nitrides [42] have been developed and studied for potential applications in many fields. Their uniquely high aspect ratios (atomically thin) allow such materials to be assembled into ultrathin membranes exhibiting a high permeation flux [43]. Among the 2D materials, graphene-based membranes have attracted a vast amount of interest due to their high thermal and mechanical stability, along with the residing functional groups, allowing a further tuning of the membrane structure. Thus, single-layer or graphene laminates were formed onto porous membrane supports to develop thin-film composite membranes [44,45,46], or graphene was employed as the filler material in mixed-matrix membrane (MMM) fabrications [47].

In this work, for the first time, the recent progress on graphene-based membranes for H_2_ separation was comprehensively reviewed, in contrast to previous reviews that broadly cover general gas separations using graphene membranes [48,49,50]. First, this review begins with the introduction of three commonly adopted membrane designs, namely single-layer graphene, multi-layer graphene laminates and graphene-based composite membranes or MMMs. This is followed by an overview on the recent progress in graphene-based membranes for H_2_ separation. The H_2_ separation performances are plotted together with the upper bound limits for H_2_/CO_2_, H_2_/N_2_ and H_2_/CH_4_ separations for effective benchmarking. The properties of the selected gases are summarized in Table 1 for reference. The studies on molecular simulation and modeling are also discussed in this review. Finally, the challenges, potential and future directions of graphene-based membranes for H_2_ separation are discussed in the conclusions section.

## 2. Graphene-Based Membrane

**Figure 1:**
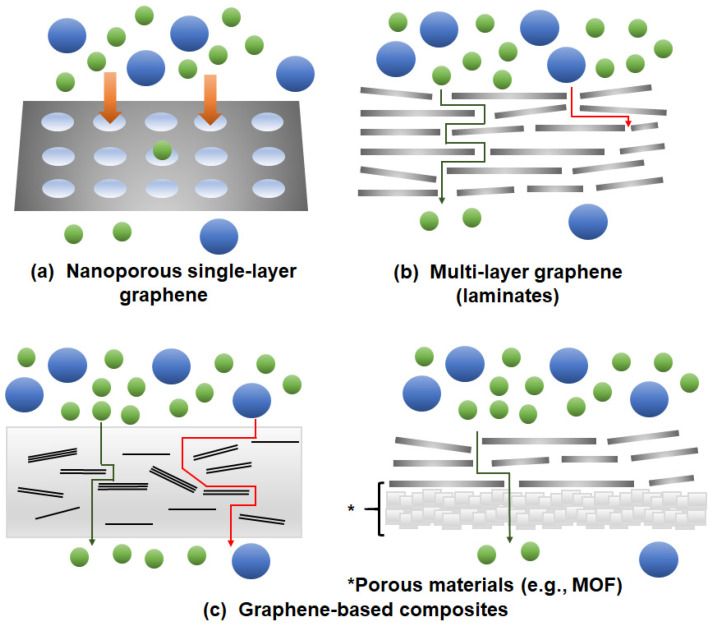
Possible membrane configurations that can be developed with the use of graphene: (**a**) Nanoporous single-layer graphene, (**b**) multi-layer graphene (laminates) and (**c**) graphene-based composites. MOF: metal-organic frameworks.

In this section, a brief overview on the various graphene membrane configurations, namely single-layer, multi-layer and graphene-based composites (Figure 1), is presented. We also invite readers to refer to the following references [53,54,55] in order to obtain a better insights on the development on graphene-based membranes under various configurations.

### 2.1. Single-Layer Graphene

**Figure 2:**
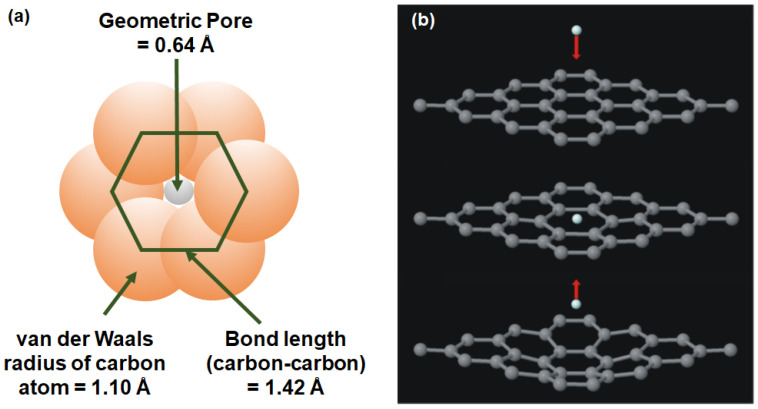
(**a**) Comparison among the van der Waals radius of a carbon atom (1.10 Å), carbon-carbon bond length (1.42 Å) and geometric pore (0.64 Å). It can be observed that the pores are too small to allow the penetration of gases. Reproduced with permission from Reference [58], copyright 2013 Elsevier. (**b**) Reflection of the He atom from the graphene surface upon transport through the available pores on nondefective single-layer graphene. Reprinted with permission from Reference [59], copyright 2008, AIP Publishing LLC.

Graphene is a 2D nanomaterial in which carbon atoms are arranged in a hexagonal lattice via sp^2^ hybridization. Graphene possesses extraordinary thermal, electrical and mechanical properties due to the presence of long-range π-conjugation throughout the structure. Moreover, the chemical property of graphene can also be tuned so as to develop reduced graphene oxide (rGO) and graphene oxide (GO). In general, defect-free nonperforated single-layer graphene is not permeable to gas molecules. This is attributed to the delocalized π-electron clouds in the aromatic rings that blocks the penetration of any molecules [56,57]. As shown in Figure 2a, the geometric pore (0.64 Å) in the single-layer graphene that is calculated from the van der Waals radius of carbon (1.10 Å) is smaller than the kinetic diameter of helium (Table 1) [53,58]. Apart from this, the impermeability of gases in single-layer graphene was also verified with the aid of molecular dynamics (MD) simulation and density functional theory (DFT). The energy barrier that is required for monoatomic molecules (e.g., helium (He)) to pass through a nondefective graphene layer is calculated to be 18.8 eV based on the local density approximation (LDA). Given that the kinetic energy of the He atom is calculated to be 18.6 eV, it is not possible for He to permeate through the single-layer graphene sheet (Figure 2b) [59]. Thus, the creation of subnanometer pores that can serve as gas transport channels in the single-layer graphene has been the major research focus, which will be elaborated further in Section 3.1.1. It is noteworthy that this type of membrane is not applicable in practical processes owing to its poor scalability.

### 2.2. Multi-Layer Graphene

**Figure 3:**
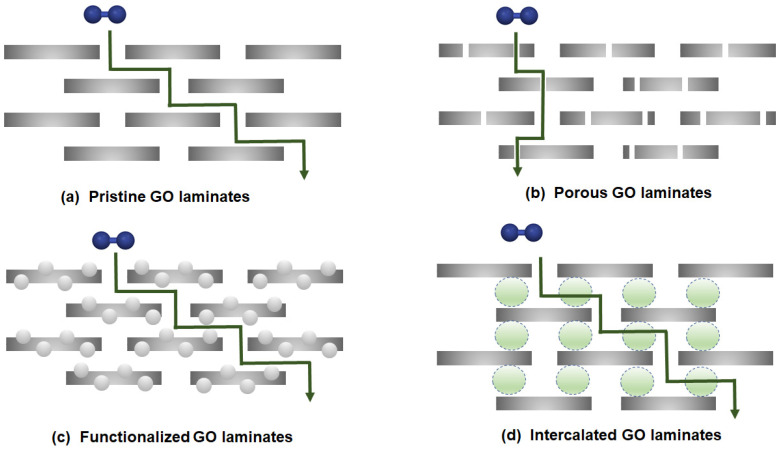
Possible configurations of graphene laminates: (**a**) pristine graphene oxide (GO) laminates, (**b**) porous GO laminates, (**c**) functionalized GO laminates and (**d**) intercalated GO laminates. Reproduced with permission from Reference [54], Creative Commons License CC BY 4.0.

In comparison to a single-layer graphene membrane, the creation of multi-layer stacked graphene is considered to be technically viable, as multi-layer graphene laminates with high-quality and integrity can be readily developed less stringently in membrane design. To date, the utilization of multi-layer graphene for gas separation commonly involves the synthesis of GO, which is one of the main derivatives of graphene. This is attributed to its well-established synthesis method (graphite oxidation), as well as the presence of abundant functional groups on the basal and edge of graphene planes (e.g., epoxy, carboxyl and hydroxyl). As these hydrophilic functional groups allow GO to be dispersed stably in polar solvents such as water, GO can be processed into thin films and membranes. However, the development of a defect-free continuous film is necessary in order to prevent the undesirable nonselective transport of gases. This is typically accomplished by increasing the thickness of GO laminates. Nevertheless, such action leads to a compromise in gas permeability (or flux), as depicted by the Hagen-Poiseuille equation [60]. Besides, according to the calculations by Nielsen [61], if the graphene sheets are oriented perpendicular to the permeation direction, the diffusion length is expected to be 1450 times greater, with respect to the thickness of the graphene laminates (Figure 3a). Thus, various strategies such as porous graphene laminates (Figure 3b), functionalized GO laminates (Figure 3c) or intercalated GO laminates (Figure 3d) have been proposed to tune the resulting gas separation performance. The details about the effects of various fabrication processes on H_2_ separation performance will be discussed in Section 3.1.2.

### 2.3. Graphene-Based Composites

Similar to other commonly reported porous materials (e.g., zeolites, MOFs and MOPs), graphene can serve as the filler in composite membranes or MMMs [47,62,63,64]. By utilizing the interlayer channels that are present in graphene laminates, the selective molecular transport of targeted gas can be realized. Apart from this, it has been reported that incorporation of graphene into the polymer matrix can improve the mechanical properties as compared to the pure polymeric membrane. For instance, the incorporation of 10 wt% GO into a Matrimid^®^ 5218 membrane increased the Young’s modulus and tensile strength by 12% and 7%, respectively [47]. In a similar study, the incorporation of 5 wt% GO in an ODPA-TMPDA (4,4′-oxydiphthalic anhydride and 2,4,6-trimethyl-*m*-phenylenediamine) membrane was able to improve both the Young’s modulus (8%) and tensile strength (11%), respectively [65]. Such behavior has been attributed to a strong chemical interaction between GO rich in functional groups and the polymer matrix.

Moreover, GO has also been utilized as the building block in creating highly porous composite materials with 3D architectures. Typically, MOFs are selected as the other component in such a 3D architecture, as the functional groups in GO allow favorable interactions with ligands in MOFs. As a result of the integration of 2D GO, additional gas adsorption sites (i.e., microporosity) can be generated in the composite. This is exemplified by the increase in accessible surface area (evaluated using Brunauer-Emmett-Teller (BET) surface area based on a N_2_ physisorption measurement at 77 K) [66], as shown in Table 2. Indeed, several MOF/GO composites that were not limited to ZIF-8/GO [67], HKUST-1/GO [68], MOF-5/GO [69] and NiDOBDC/GO [64] were successfully synthesized. It was reported that MOFs with a smaller crystal size (i.e., nanocrystals) are preferred to generate a more uniform 3D structure, leading to a substantial increase in the accessible surface area (Table 2). In general, the 3D architectures can be constructed through two major approaches: (1) physical mixing of MOF and GO or (2) in-situ growth of MOF on the surface of GO. The performance of graphene-based composites in H_2_ separation will be elaborated on in Section 3.1.3.

## 3. Performance of a Graphene-Based Membrane

### 3.1. Investigation of the Membrane’s Performance in H_2_ Separation

Table 3, Table 4 and Table 5 summarize the performances of graphene-based membranes in various gas separations involving H_2_ (H_2_/CO_2_, H_2_/N_2_ and H_2_/CH_4_), as reported in the literature. These tables show the properties of the selective layer (material and membrane thickness), type of membrane support, permeation testing conditions and the gas permeation properties (in terms of H_2_ permeance and gas selectivity). For the ease of comparison, H_2_ permeances of all membranes are reported in gas permeation unit (GPU), with 1 GPU = 10^−6^ cm^3^ (STP) cm^−2^ s^−1^ cmHg^−1^. In general, most graphene-based membranes reported are in the integrally skinned asymmetric (ISA) structure, whereas symmetric (dense) membranes are formed if graphene is served as the filler dispersed in polymer matrices [7].

#### 3.1.1. Single-Layer Graphene

**Figure 4:**
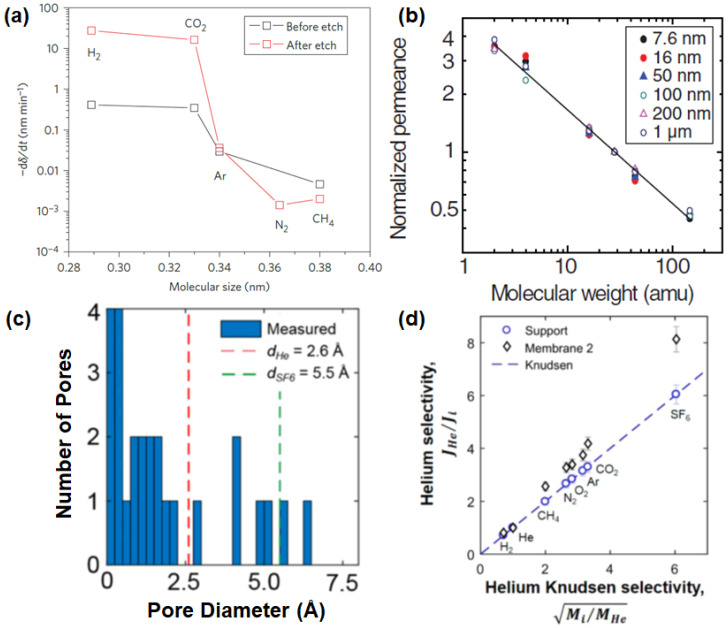
(**a**) Average deflection (-dδ/dt) against molecular size for a bilayer graphene membrane (Bi-3.4 Å). Reprinted with permission from Reference [108], copyright 2012, Nature Publishing Group. (**b**) The trend of gas permeability (permeance of H_2_, He, CH_4_, N_2_, CO_2_ and SF_6_ that is normalized with N_2_ permeance) for single-layer graphene with various average pore diameters. The solid line on the graph indicates the inverse square root of mass dependence (illustration of Knudsen selectivity). Reprinted with permission from Reference [79], copyright 2014, American Association for the Advancement of Science. (**c**) Pore size distribution of single-layer graphene measured with the aid of scanning transmission electron microscopy (STEM). (**d**) Ratio of the helium flow rate to those of other gases that is plotted against Knudsen selectivity. Reprinted with permission from Reference [89], copyright 2017, American Chemical Society.

As mentioned in Section 2.1, the unique atomic thickness of graphene may open up a chance to achieve the highest flux, although a defect-free graphene sheet is impermeable to all gases. The successful formation of a single-layer graphene membrane typically involves two critical steps: (1) the transfer of large-area graphene onto a desired porous substrate without appreciable tears and cracks and (2) the creation of subnanometer pores with a narrow pore size distribution. The investigation of single-layer porous graphene for selective molecular transport was first conducted with the use of UV-induced oxidative etching of a graphene sheet on a porous SiO_2_ support. As reported by Koenig et al. [108], even though the gas permeance was not calculated in a precise manner, an investigation on the leak rate of porous the graphene formed by the etching process showcased its potential in H_2_/N_2_ and H_2_/CH_4_ separations, as shown in Figure 4a. This is subsequently followed by the utilization of focused ion beam (FIB) milling to generate pores on a single-layer graphene that was attached on a freestanding SiN_x_ support. In this work, the transfer of dual-layer graphene sheets rather than single-layer graphene sheets was conducted in order to improve the stability of the free-standing graphene. The pores in two different ranges could be obtained with the use of gallium (Ga^+^) and helium (He^+^) ions, with the resulting pore sizes ranging from 10 nm to 1000 nm. With the overall porosity of a mere 4%, an extraordinarily high gas permeance could be achieved while the selectivity stayed at the Knudsen selectivity (Figure 4b) [79].

To overcome the limitation of the previous work, a porous graphene with pores in the subnanometer range needed to be developed, leading to a molecular sieving beyond the Knudsen selectivity. In the study conducted by Boutilier et al. [89], a single-layer graphene possessing a large amount of permeable pores was produced at the centimeter scale. An observation by aberration-corrected scanning transmission electron microscopy (STEM) after bombarding gallium ions onto the surface of graphene revealed that the developed membrane possessed pores that can discriminate He (red-dotted line) from SF_6_ (green-dotted line) (Figure 4c). However, cracks could be formed in the single-layer graphene during the transfer onto a porous support, resulting in a separation performance that was merely close to the Knudsen selectivities for H_2_/CH_4_ (3.14) and H_2_/CO_2_ (5.16), respectively (Figure 4d). The possibility of the crack formation can be mitigated by employing the wet-transfer technique. This method allows an effective transfer of single-layer nanoporous graphene onto a wide range of substrates without jeopardizing the intrinsic selectivity. In addition, an oxidative etching technique (e.g., oxygen, oxygen plasma and ozone) was able to create pores under the resolution of a subnanometer range [96,100], leading to a gas separation performance of single-layer graphene surpassing the Knudsen selectivity of the desired gas pairs (e.g., H_2_/CH_4_). A recent study on developing a single-layer graphene membrane with the use of chemical vapor deposition (CVD) was reported by Rezaei et al. [107]. In this work, the copper (Cu) foil was used as the catalytic substrate. It has been observed that the H_2_/CH_4_ separation performance of single-layer graphene is heavily influenced by the intrinsic purity and uniformity of the Cu foil. Thus, to improve the potential feasibility of utilizing Cu foil in the large-scale fabrication of single-layer graphene, it was proposed to anneal the Cu foil at a temperature close to its melting point. Such a process reduces the overall surface roughness of the Cu foil, leading to a uniform growth of the graphene layer. In such a case, an improvement of the H_2_/CH_4_ selectivity by 1-to-1.5-fold can be achieved.

#### 3.1.2. Multi-Layer Graphene

As elaborated in the previous section, several undesirable bottlenecks are expected in the development of defect-free single-layer graphene membranes. Particularly, it is still technically challenging to transfer a graphene layer onto a porous substrate while keeping a good integrity without defects and leaks [101]. Thus, alternatively, the fabrication multi-layer graphene laminates on porous substrates has been a major focus in the H_2_ separation process.

In general, one of the most common approaches in multi-layer graphene membrane fabrication is the vacuum filtration method. In the study by Romanos et al. [81], the membrane performance was investigated while varying several parameters, such as the filtration rate (controlling the downstream pressure), volume of GO suspension (thickness of the selective layer) and surface chemistry (GO and rGO). From the gas permeation data, a slower filtration rate led to a membrane showing molecular sieving characteristics. This is because a faster filtration rate generates a haphazard arrangement of GO stacks, resulting in a larger porosity. Besides, the gas permeation results indicated that a sufficient amount of GO suspension is required develop a highly selective membrane. Lastly, rGO often created undesirable membrane defects during the fabrication, as the reduction of GO gave a negative impact on the dispersibility in both water and organic solvent due to a decrease in hydrophilicity. The formation of rGO agglomerates are reported to be irreversible in spite of using a sonication process, which is typically adopted as an action to minimize aggregation between GO sheets [90,102,109].

**Figure 5:**
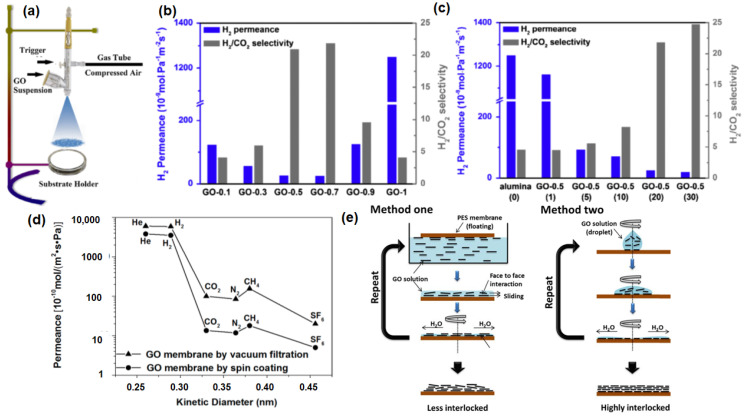
(**a**) Schematic illustration of the spray evaporation process. Reprinted with permission from Reference [94], copyright 2018, Elsevier. (**b**) H_2_ separation performance of GO membranes with variations of the evaporation rate (0.1 to 1, based on the mass fraction of ethanol in an aqueous solution). (**c**) H_2_ separation performance of GO membranes with variations of the spraying time (time in this context refers to the number of spray coatings conducted, which is indicated in parentheses). Reprinted with permission from Reference [85], copyright 2017, Elsevier. (**d**) Comparison of the pure gas permeance of the GO membranes by spin coating and vacuum filtration. Reprinted with permission from Reference [83], copyright 2016, American Chemical Society. (**e**) Comparison between the GO membranes prepared by method one (contacting the polyethersulfone (PES5) membrane with the GO solution, followed by spin coating) and method two (spin casting). Reprinted with permission from Reference [77], copyright 2013, American Association for the Advancement of Science.

The microscopic structure of the GO membrane fabricated via vacuum-filtration is reported to be unpredictable, even though the filtration rate is controlled. This is due to the fact that the unavoidable evaporation of solvent molecules results in the formation of the nonuniform (random) packing of GO sheets [110]. Owing to the absence of external forces, a heterogeneous layer with a loop structure (i.e., wrinkling) can be formed [111]. Furthermore, the vertical pulling force by vacuum filtration does not ensure a well-ordered horizontal alignment of GO nanosheets onto a porous membrane support [83]. To minimize such effects, Guan et al. [85] and Ibrahim et al. [94] fabricated GO laminate membranes by a spray evaporation-induced self-assembly approach (Figure 5a). Contrary to the conventional evaporation method, the spraying process utilizes ultra-small droplets, leading to a higher evaporation area. Apart from this, the capillary action between the substrate and GO, as well as hydrogen bonding (or van der Waals forces) between GO nanosheets during each spraying step, give rise to the separation layer with uniform thickness. Further investigation on the spraying conditions, including evaporation rate (Figure 5b) and spraying time (Figure 5c), was also conducted. As shown in Figure 5b, an excessive reduction of evaporation time led to the formation of wrinkles and random deposition of GO laminates, resulting in a high H_2_ permeance with a low H_2_/CO_2_ selectivity. A longer spraying time is typically required to achieve a uniform coverage of the substrate with GO laminates, resulting in a reasonably high H_2_/CO_2_ selectivity (Figure 5c).

**Figure 6:**
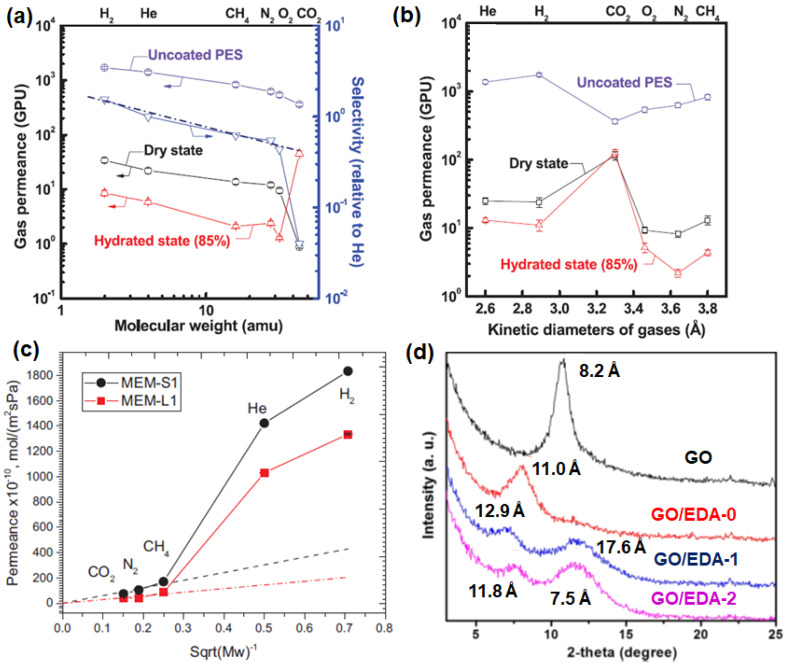
(**a**) Gas permeation of the GO membranes (method one) as a function of molecular weight (the black dash line corresponds to the ideal Knudsen selectivity). (**b**) Gas permeation of the GO membranes (method two) as a function of the kinetic diameter. Reprinted with permission from Reference [77], copyright 2013, American Association for the Advancement of Science. (**c**) Pure gas permeation of MEM-S1. Reprinted with permission from Reference [95], copyright 2018, Elsevier. (**d**) X-ray diffraction (XRD) profiles of GO, GO/EDA-0, GO/EDA-1 and GO/EDA-2 (0, 1 and 2 refer to the crosslinking time in hours). Reprinted with permission from Reference [90], copyright 2018, American Chemical Society.

The fabrication of multi-layer GO laminates can also be conducted with the coating techniques. In general, the performance of GO membranes that were fabricated from spin coating is reported to be higher than that of membranes synthesized by the vacuum filtration method (Figure 5d), due to the creation of a more uniform structure without the formation of wrinkles. In the study by Kim et al. [77], GO coating was conducted by using both spin coating (method one) and spin casting (method two), as shown in Figure 5e. GO membranes fabricated by method one showed less interlocked structures in comparison to method two. Thus, not surprisingly, the separation performance of the GO membrane fabricated by method one is correlated to Knudsen selectivity (illustrated by the dashed line in Figure 6a). This is because the presence of edge-to-edge repulsion in GO nanosheets causes the formation of an island-like assembly in the GO laminates. Nevertheless, due to the potential formation of hydrogen bonding between CO_2_ and the polar groups in GO, a much lower CO_2_ permeance and higher H_2_/CO_2_ selectivity were reported (Table 3). On the other hand, in method two, both attractive and repulsive forces on GO nanosheets can be foreseen, as both casting and spinning processes occur simultaneously, leading to a highly interlocked structure in the resulting GO laminate. Consequently, a CO_2_-selective membrane (rather than a H_2_-selective membrane) can be successfully made by method two. Notably, an introduction of humidified feed gas resulted in a sacrificial decrease in gas permeance due to the blockage of the permeation channel between GO layers by condensed water molecules (Figure 6a,b). However, such a phenomenon was not prominent when CO_2_ was the dominant permeate gas, presumably due to the favorable interactions between CO_2_ and the condensed water molecules. As such, the GO membranes developed by method one (Figure 6a) were found to be CO_2_-selective rather than H_2_-selective.

Apart from this, the performance of GO membranes can be altered by employing GO with different flake sizes. In general, the in-house synthesis protocol typically produces GO sheets with small and irregular lateral dimensions, since such synthesis involves the exfoliation of GO layers based on a sonication process. Thus, with the variation of the sonication period, a wide distribution of the flake size can be caused [95]. This behavior often resulted in a nonuniform GO coverage or the formation of appreciable defects. To mitigate such a problem, a freeze-thaw exfoliation was utilized, leading to GO nanosheets of large dimension (~13 μm) [83]. The GO membranes fabricated by such large GO flakes showed a high selectivity due to the formation of a more tortuous diffusion path (Figure 6c), which was also observed in a recent work [95].

Another way to improve the separation performance of GO membranes is the alteration of the interlayer spacing (or *d*-spacing) of GO laminates. This spacing can be served as “slits” affecting the molecular transport in GO membranes [63]. Various intercalators that are not limited to metal ions, cations, amines and polymers [112,113,114] can be adopted into the interlayer spacing of the GO nanosheets through a crosslinking process. For example, Lin et al. [90] used ethylenediamine (EDA) as the crosslinker for the fabrication of GO membranes, as the carboxylic acid groups (–COOH) on GO allowed an effective attachment of EDA onto the GO sheets. The crosslinking in-between EDA and GO can be conducted while varying the reaction time (GO/EDA-0, GO/EDA-1 and GO/EDA-2). The insertion of EDA in-between the GO layers increased the interlayer spacing from 8.2 Å to 11 Å (Figure 6d). This resulted in a 166% increase in the H_2_ permeance but a 33% decrease in the H_2_/CO_2_ selectivity with respect to the performance of the pure GO membrane. On the other hand, due to the presence of smaller *d*-spacing with the increase in crosslinking time, a substantial decrease in the H_2_ permeance (−47%) with an increase in H_2_/CO_2_ selectivity (15%) was observed for the GO/EDA-1 membrane. A similar result was also reported by Cheng et al. [103]. In this study, it was reported that an optimal amount of crosslinker (cysteamine in this study) was required to prevent the formation of extraordinary large interlayer spacing, which decreased the overall H_2_/CO_2_ selectivity. In a recent study by Chuah et al. [41], different cations (Co^2+^ and La^3+^) were intercalated in between GO nanosheets to tune the interlayer spacing. The gas separation performance indicated that the incorporation of cations is feasible to increase the H_2_ permeance due to the increase in interlayer spacing (8.5 Å for GO-Co^2+^ and 8.8 Å for GO-La^3+^) in comparison to pure GO (8.8 Å). The H_2_/CO_2_ selectivities of GO-Co^2+^ and GO-La^3+^ are reported to be considerably higher than that of pure GO, which is potentially attributed to the presence of chemical interactions between metal ions (Co^2+^ and La^3+^) and CO_2_ molecules.

Furthermore, the interlayer spacing of GO nanosheets can be varied by adjusting the synthesis method of GO. The modified Hummers’ method (GO-H) is most common in the synthesis of GO flakes due to the rapid graphite oxidation [115]. However, it is considerably challenging to decrease the interlayer spacing of the GO membrane to a value smaller than 8.8 Å for this case. Therefore, the development of GO membranes with the use of GO nanosheets that are synthesized from the modified Brodie’s method (GO-B) was explored by Ibrahim et al. [116]. It was observed that GO-B membranes can be fabricated with a much smaller interlayer spacing (6.0 Å) in comparison to GO-H, leading to more effective molecular sieving. From the gas permeation testing [98], the increases in H_2_/CO_2_, H_2_/N_2_ and H_2_/CH_4_ selectivities by 129%, 91% and 76%, respectively, were reported when GO-B was employed in the membrane fabrication instead of GO-H. However, it should be noted that this synthesis protocol is more complicated and tedious as compared to the Hummer’s method, since repetitive graphite oxidation is required (typically about three ~ four times) to obtain the desired product (C/O ratio > 2).

Last, but not least, the fabrication of large-scale multi-layer graphene laminate membranes has been demonstrated. The flat sheet configuration that is commonly reported in the literature (summarized in Table 3, Table 4 and Table 5) is not a popular choice in practical gas separations due to the limited membrane area per unit volume. Rather, the hollow fiber or capillary configuration that provides a high surface area-to-volume ratio is more frequently chosen. In this context, a dip coating by soaking hollow fiber membrane supports into a GO dispersion has been tried, with the lumen side being connected to a mild vacuum to mimic the vacuum filtration method, as in flat sheet membrane fabrication. Such an approach is often termed as the vacuum suction method [86,88,91,97,99]. Nevertheless, the optimization of selective layer thickness is known to be difficult, since an increase in the GO coating time may not result in a linear increase in the thickness of GO laminates. This is because the deposited GO layers serve as undesirable resistances inhibiting the additional stacking of GO sheets on the hollow fiber supports [88].

#### 3.1.3. Graphene-Based Composites

**Figure 7:**
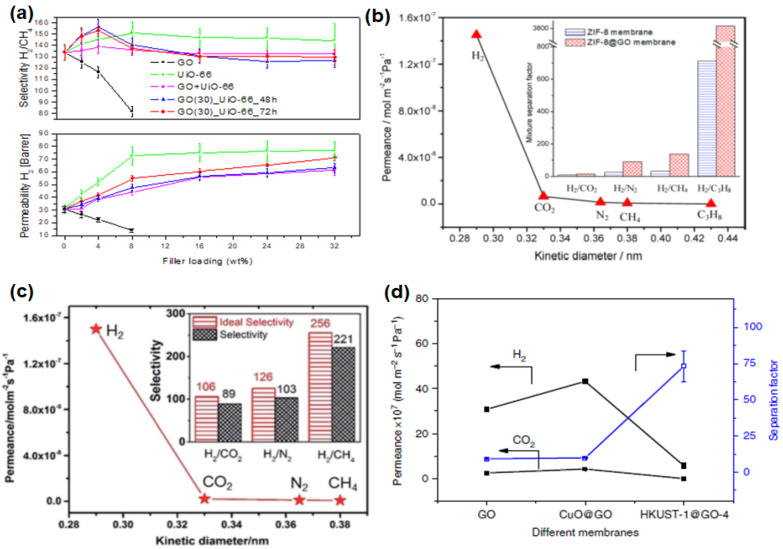
(**a**) Performance of the mixed-matrix membrane (MMM) with the incorporation of GO, UiO-66 and UiO-66/GO composites (GO_UiO-66) and the physical blending of GO and UiO-66 (GO + UiO-66). Reprinted with permission from Reference [105], copyright 2017, Elsevier. (**b**) Single-gas permeance (at 250 °C) of the ZIF-8@GO membrane as a function of the kinetic diameter. The inset includes the selectivities of the ZIF-8 and ZIF-8@GO membranes. Reprinted with permission from Reference [80], copyright 2014, American Chemical Society. (**c**) Single-gas permeance of the GO-Zn_2_(bim)_4_ membrane. The inset includes the ideal and mixed-gas selectivities. Reprinted with permission from Reference [93], Creative Commons Attribution CC BY 4.0. (**d**) Mixed-gas H_2_/CO_2_ separation performance of the GO membrane, CuO@GO membrane and HKUST-1@GO-4 membrane. Reprinted with permission from Reference [92], Creative Commons Attribution CC BY 4.0.

The potential utility of GO in altering the H_2_ separation performance of polymer membranes (in polysulfone and Matrimid^®^ 5218) was investigated by Castarlenas et al. [105]. Based on the gas permeation data, at the GO loadings of 4 wt% and 8 wt%, undesirable decreases in both H_2_ permeability (c.a. 140% and 100%, respectively) and H_2_/CH_4_ selectivity (c.a. 50% and 38%, respectively), were observed (Figure 7a). This behavior is attributed to the nonporous nature of GO creating tortuous diffusion pathways in the membrane [117]. Besides, the decrease in H_2_/CH_4_ selectivity is plausibly attributed to a higher polarizability of CH_4_ [51] than H_2_ (Table 1), leading to a more favorable sorption of CH_4_ in the membrane over H_2_. Rather, these membranes were found to be more suitable in CO_2_/CH_4_ separation, as evidenced by a gradual increase in mixed-gas selectivity with the increase in GO loading [47,65]. Hence, the incorporation of a GO/MOF composite filler has been tried to improve the H_2_ separation performance, since such a composite filler not only has a high porosity but, also, minimizes the aggregation of MOFs [30,118]. Such an idea was verified by comparing the H_2_/CH_4_ separation performance of MMMs comprising (1) UiO-66/GO composites and (2) the physical blending of UiO-66 and GO [105]. Based on the gas permeation results, enhancements in both H_2_ permeability and H_2_/CH_4_ selectivity were observed for the case of UiO-66/GO composites (Figure 7a), which also showed a good interfacial interaction between the filler and polymer matrix. In contrast, the physical blending was found to be less effective in improving H_2_/CH_4_ selectivity.

One of the major advantages of constructing a 3D architecture membrane with MOF and GO was reported to be a reduction of membrane defects. As mentioned in the previous section, wrinkles can be formed in GO laminates that are fabricated via vacuum filtration. Besides, the creation of a smooth continuous GO layer can be challenging if the solvent used does not form a good GO dispersion. Thus, Huang et al. [80] developed a bi-continuous ZIF-8@GO membrane by using layer-by-layer assembly. GO in this membrane serves as the sealant to fill the gaps in the ZIF-8 membrane. Owing to the presence of capillary forces between GO and ZIF-8, the gas molecules are expected to diffuse dominantly through the micropores in ZIF-8, leading to an extraordinary high H_2_ permeance and decent H_2_/CO_2_, H_2_/N_2_ and H_2_/CH_4_ selectivities (Figure 7b). The ZIF-8@GO composite membranes without appreciable defects were also made by sealing the gaps in GO membranes with ZIF-8 crystals grown in the defective region [82]. Gas permeation testing revealed that the deposition of ZIF-8 onto defective graphene layers can improve the H_2_ permeance by 5.4 times (Table 5). In a subsequent study by Jia et al. [87], the deposition of UiO-66-NH_2_ (presynthetic modification UiO-66 with a 2-aminoterephtalitc acid ligand) and GO was conducted in two different ways: (1) simultaneous deposition of UiO-66-NH_2_ and GO onto the support (GOU@S) and (2) layer-by-layer deposition of UiO-66-NH_2_ and GO onto the support (GO/U@S). The performances of both GOU@S and GO/U@S in H_2_/CO_2_ and H_2_/N_2_ separations were improved substantially as compared to GO@S due to the presence of hydrogen bonding and electrostatic force that eliminated the formation of nonselective voids. Nevertheless, the performance of GOU@S was more promising than GO/U@S due to the better integrity of the overall membrane structure (Figure 7c).

In general, ZIF-8 and UiO-66 (and its derivative) are commonly used in membrane fabrication due to their scalable synthesis and decent stability under humid conditions. Nevertheless, other MOF-based composites have also been synthesized using HKUST-1 particles and Zn_2_(bim)_4_ nanosheets, as reported by Kang et al. [92] and Li et al. [93], respectively. Both membranes are developed based on similar protocols. First, a thin layer of metal oxide nanoparticles was prepared on the surface of a tubular support. This is followed by the addition of GO and the respective ligand to conduct an in-situ transformation into MOF@GO composites. The membrane comprising Zn_2_(bim)_4_ nanosheets exhibited a molecular sieving property (Figure 7d), as evidenced by high H_2_/CO_2_, H_2_/N_2_ and H_2_/CH_4_ selectivities in Table 3, Table 4 and Table 5 [93]. The HKUST-1 crystals are also able to improve the H_2_/CO_2_ selectivity from 12.2 to 73.2. However, a sharp decrease in H_2_ permeance was accompanied, which is not commonly encountered in previous studies. Such a deviation could be attributed to the uneven distribution or arrangement of HKUST-1 bulk crystals in comparison to Zn_2_(bim)_4_ nanosheets. Nevertheless, subsequent tuning of the selective layer through the variation of filtration cycles proved that H_2_/CO_2_ separation performance can be optimized by controlling the thickness of the selective layer.

Meanwhile, other materials can also be used as the pillar to synthesize GO-based composites. For example, in a recent study conducted by Guo et al. [104], hydroxy sodalite (SOD) nanocrystals were impregnated into GO layers to design a H_2_-selective membrane. The incorporation of SOD nanocrystals increased the H_2_/CO_2_ selectivity from 9 to 105, due to the small pore aperture (2.9 Å) of SOD. Nonselective defects were not formed due to the strong hydrogen bonding between SOD and GO, which was confirmed by the shifting of the O–H band from 1641 cm^−1^ to 1600 cm^−1^ in the Fourier-transform infrared spectroscopy (FTIR) analysis.

### 3.2. Molecular Simulation and Modeling

#### 3.2.1. Single-Layer Graphene

**Figure 8:**
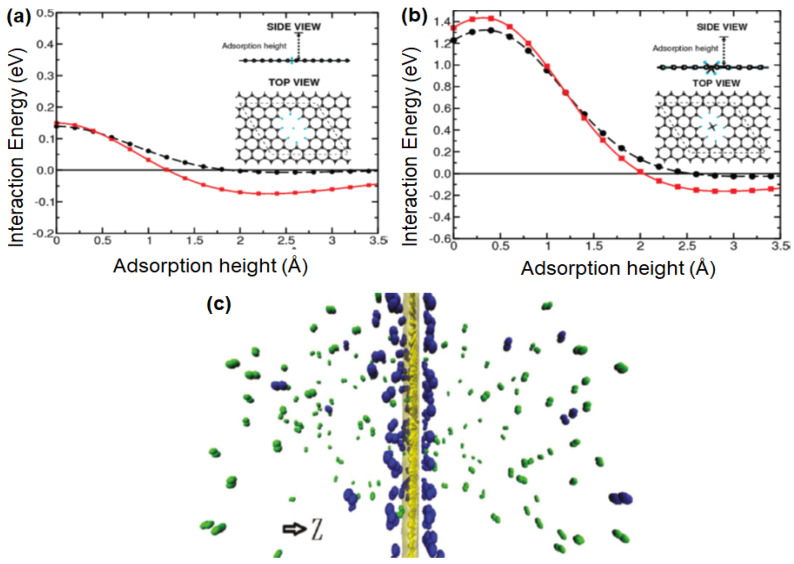
Interaction energy between (**a**) H_2_ and (**b**) CH_4_ and all-hydrogen passivated porous graphene as a function of the adsorption height. The red and black lines are referring to density functional theory (DFT) calculations conducted by the van der Waals density functional (vdW-DF) and Perdew, Burke and Erzenhoff functional (PBE), respectively. Reprinted with permission from Reference [120], copyright 2009, American Chemical Society. (**c**) Gas distribution of N_2_ (green) and H_2_ (blue) on the porous graphene surface. Reprinted with permission from Reference [121], copyright 2013, American Chemical Society.

As discussed in Section 2.1, the pore formation is of paramount importance in order to induce the transport of atoms or molecules through graphene layers [119]. Thus, several computational studies to investigate the effect of pore size on the separation performance have been reported. The propensity of single-layer graphene in H_2_ separation was first studied by Jiang et al. [120] with the aid of DFT. Such calculations can be employed to determine the energy barriers for the transport of gaseous molecules through the porous graphene. As shown in Figure 8a,b, the energy barriers for H_2_ and CH_4_ transports through a porous graphene with 2.5-Å pores are calculated to be 0.22 and 1.60 eV, respectively. Considering the fact that 0.22 eV of the energy barrier for H_2_ can be readily overcome under ambient conditions, a high H_2_/CH_4_ selectivity is predicted.

Subsequently, the performance of porous graphene in H_2_/N_2_ separation was studied while varying the sizes and shapes of the pores [121], with the use of MD simulation. MD is generally useful to predict the physical movement of molecules as a function of time. Two different optima corresponding to the maximum H_2_/N_2_ and N_2_/H_2_ selectivities could be observed when the pore size was gradually increased. This is because the adsorption of N_2_ on the graphene’s surface is preferred over H_2_ (Figure 8c), although the size of H_2_ is much smaller than N_2_ [51]. This phenomenon resulted in a more effective diffusion of N_2_ molecules if the pore size was sufficiently larger than the size of N_2_. Meanwhile, the surface property of porous single-layer graphene may govern the resulting gas transport behavior, as reported by Drahushuk et al. [122] and Tao et al. [123]. For example, CO_2_, which possess the highest polarizability (Table 1) as compared to commonly reported gases (N_2_, H_2_, He and CH_4_), is strongly bound to the graphene surface rich in polar functionality, thus resulting in a poor CO_2_ diffusivity and a high H_2_/CO_2_ selectivity [101]. However, it should be noted that the practical feasibility of single-layer graphene is typically hampered by its strong susceptibility to defect formations. For instance, the diffusion barrier for CH_4_ permeation through a single-layer graphene decreases to 0.02 eV at the pore size of 3.8 Å and 0 eV at 5.0 Å, respectively, from 1.60 eV at 2.5 Å [120].

#### 3.2.2. Multi-Layer Graphene

**Figure 9:**
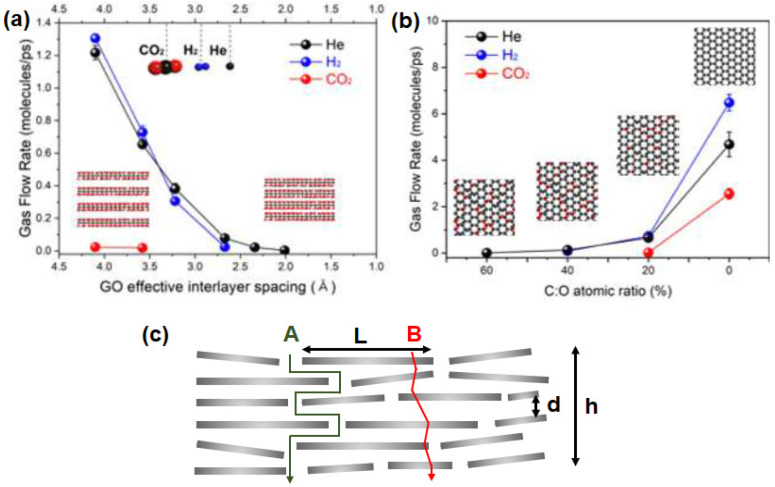
(**a**) Effect of the change of the interlayer spacing (interlayer carbon-to-carbon system is varied from 4.75 Å to 7.50 Å) on the gas flow rate (gas permeance). (**b**) Effect of the variation of the oxidation degree (atomic ratio between C and O, in which a higher C:O refers to a higher oxidation degree) on the gas flow rate. Reprinted with permission from Reference [102], copyright 2020, Elsevier. (**c**) Gas transport model through a multi-layer graphene membrane. Adapted with permission from Reference [95], copyright 2018, Elsevier.

Computational work on multi-layer graphene membranes typically studied the three different parameters, namely interlayer spacing between GO layers, oxidation degree and permeation conditions (temperature and pressure). In general, an increase in the interlayer spacing can increase the permeance across all gas molecules [102], as demonstrated in Figure 9a. This is evidently observed for the case of CO_2_, in which a noticeable flow is observed at interlayer spacings higher than 3.50 Å [51]. However, a decrease in H_2_ permeance can often be observed in mixed-gas permeations through a sufficiently large interlayer spacing due to the hindrance by competing molecules [124]. On the other hand, an increase in the oxidation degree (Figure 9b) leads to a substantial decrease in gas permeance. Such an effect is particularly noticeable for molecules with larger kinetic diameters owing to the increased steric hindrance by the GO surface [124]. It is also reported that the gas transport behavior in GO laminates can be influenced by the permeation conditions. In general, an increase in the simulation temperature resulted in an increase in the kinetic energy of the permeating H_2_ molecule, leading to a rapid H_2_ transport through the interlayer spacing of GO layers. Likewise, an increase in the operating pressure increases the collision frequency of the H_2_ molecules, resulting in an increase in H_2_ permeation [124].

The gas transport properties of multi-layer graphene laminates have also been predicted using the semi-quantitative approach, assuming linear adsorption for all gases [94,95]. Such an assumption is typically valid for H_2_ (in comparison to N_2_, CH_4_ and CO_2_) under the reported conditions in Table 3, Table 4 and Table 5. Considering the membrane configuration in Figure 9c, the pure gas permeance, *p*, can be expressed by Equation (1), where *h* = the overall thickness of GO, ε = porosity, τ = tortuosity, *D* = diffusivity and *S* = solubility. The subscripts A and B refer to the gas transport pathways, as depicted in Figure 9c. The diffusion rate, which is dependent on the kinetic diameter of the gas and pore size, can be calculated using Equation (2), where *ϕ* = the structure of the pore channel, *M_w_* = molecular weight of the gas and *E_d_* = activation energy for gas diffusion. In general, *E_d_* can be neglected if the ratio between the kinetic diameter of the gas molecule and the pore diameter is less than 0.6. $\tau_{A}$ can be calculated by using the ratio of the GO sheet length and thickness (L/*d*), whereas $\tau_{B}$ can be assumed to be in unity if the flow through the defective GO sheets is a uniform, straight line. However, it should be noted that the calculation of ε is highly dependent on the structures of the GO sheet and resulting membrane.

(1) $$p=\frac{1}{h}\left[\left(\frac{\varepsilon_{A}}{\tau_{A}}\right)D_{A}S_{A}+\left(\frac{\varepsilon_{B}}{\tau_{B}}\right)D_{B}S_{B}\right]$$

(2) $$D=\phi \sqrt{\frac{8RT}{\pi M_{w}}}\exp\left(-\frac{E_{d}}{RT}\right)$$

#### 3.2.3. Graphene-Based Composites

(3) $$P_{MMM}=P{\left[1-\phi_{f}+\left(\frac{1}{\frac{1}{\delta \phi_{f}}+\frac{1-\phi_{f}}{\beta \phi_{f}}}\right)\right]}^{-1}$$

(4) $$F_{index}=\ln\left(\frac{P_{MMM}}{P}\right)+\eta \ln\left(\frac{\alpha_{MMM}}{\alpha }\right)$$

The modeling studies of graphene-based composites are generally limited as compared to the theoretical models based on three-dimensional porous particles [7,125]. The gas permeation behavior of 2D fillers can be modeled using the equation proposed by Cussler [126]. In Equation (3), the gas permeability of MMM, *P_MMM_*, is related to the gas permeability of the polymeric membrane (*P*), the ratio of diffusion coefficients in the pure polymer and MMM (*δ*), aspect ratio of the flake (*β*) and volume fraction of the filler (*β)*. It is noteworthy that the presence of nonidealities (e.g., sieve-in-a-cage, plugged sieve and rigidified interface) in MMM may give different results from the prediction by this equation [7].

Apart from this, the effectiveness of graphene in composite (or MMM) membranes can be quantified with the evaluation of the filler enhancement index (*F*_index_). The *F*_index_ is an empirical metric to measure the effectiveness of a filler (graphene, in this context) in improving the gas separation performance after ruling out the effect of the polymer matrix. The calculation can be done using Equation (4), where *η* = the slope that is determined from the Robeson upper-bound limit, $\alpha_{MMM}$ = the gas selectivity of MMM and α = the gas selectivity of the polymeric membrane. Based on the calculation results, a filler in MMM can be classified into “ideal”, “exemplary”, “competent”, “moderate” or “incompetent” categories [7]. However, this relationship is not applicable to the undesirable variations of membranes, such as physical aging and plasticization [23,127,128].

### 3.3. Comparison with Upper-Bound Limits

**Figure 10:**
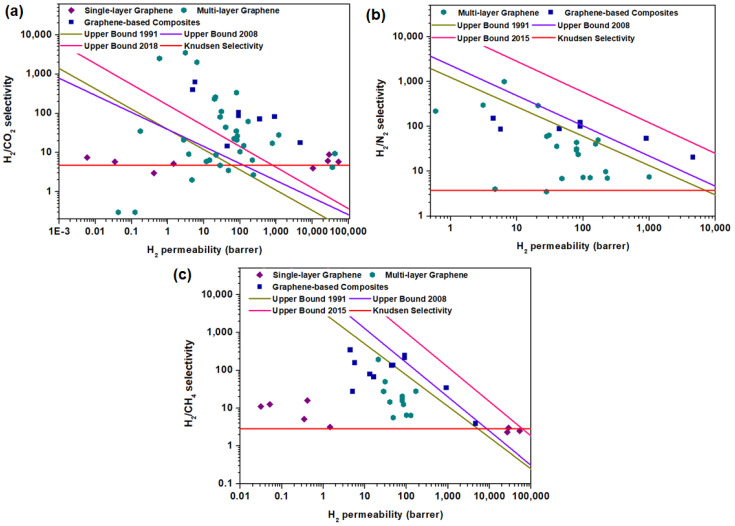
Comparison of the performances of single-layer graphene, multi-layer graphene and graphene-based composites (the data points are obtained from Table 3, Table 4 and Table 5) with the upper-bound limits for (**a**) H_2_/CO_2_, (**b**) H_2_/N_2_ and (**c**) H_2_/CH_4_ separations. The upper-bound curves are determined based on References [28,29,35,129] and summarized in Table 6.

The current performances of graphene-based membranes were benchmarked against the upper-bound limits for conventional polymeric membranes. In order to reflect the intrinsic gas separation performance of the tested membrane, it is necessary to convert H_2_ permeance into H_2_ permeability using the thickness of the selective layer. Such plots for single-layer graphene, multi-layer graphene and graphene-based composites are shown in Figure 10. The detailed parameters used to draw the upper-bound limits are provided in Table 6. Noticeably, the H_2_/CO_2_ separation performance of graphene-based membranes could surpass the upper-bound limit due to the largest margin in the molecular weights of the gas molecules leading to the highest Knudsen selectivity, in contrast to H_2_/N_2_ and H_2_/CH_4_ separations. Besides, due to the interaction between CO_2_ possessing a high polarizability and the polar groups on graphene surfaces, the diffusion of CO_2_ can be inhibited, which leads to a decrease in CO_2_ permeance and, thus, an increase in H_2_/CO_2_ selectivity. Nevertheless, graphene membranes to date have not yet been effective to perform molecular sieving based on the kinetic diameters. In particular, single-layer graphene has been reported to be quite challenging to achieve a selectivity that is higher than the Knudsen selectivity due to its susceptibility to defect formations as compared to multi-layer graphene and graphene-based composites.

## 4. Conclusions and Future Perspective

In this review, the recent progress of graphene-based membranes in the field of H_2_ separation was discussed. Single-layer porous graphene has the potential to form a highly permeable membrane, as such a configuration renders the smallest transport resistance to permeating molecules. Nonetheless, such a configuration is hampered by its low scalability and high possibility of defect formation. Hence, multi-layer graphene and graphene-based composites are studied as promising alternatives, since the fabrications of these membranes are technically viable. Eventually, the major hurdle in utilizing graphene in gas separation membranes is its capability to translate the performance when a large-scale membrane is developed for practical industrial-scale operations.

In addition to the scalability, future efforts should be given to investigating graphene-based membranes under realistic conditions. First and foremost, the measurement of gas separation performances using mixed-gas is desirable as the competition between different gas molecules has a significant impact on the gas separation performance. In this context, the H_2_ separation performance of graphene-based membranes should be measured in the presence of other components coexisting with H_2_ in the targeted real feed gases. For instance, in the typical H_2_ purification process, the presence of water vapor can give a major impact on the transport properties of H_2_ and CO_2_, since water vapor, which possesses a high polarizability and dipole moment (Table 1) [51,130], can preferentially interact with the functional groups on graphene surfaces. Secondly, graphene-membranes should be fabricated and tested in industrial module platforms, such as hollow fiber and spiral wound, the most popular configurations for gas separation applications. For example, as compared to flat films, the stacking of GO laminates can be changed in such membrane configurations, leading to different gas separation performances. To design the optimum GO membrane structure in such configurations and, eventually, achieve a high separation performance, aids from simulation and modeling studies are expected to be useful. Lastly, the stability and performance of graphene membranes should be tested under long-term operation conditions. In particular, the presence of water vapor or reactive components in the feed gas can alter the physical and chemical properties of graphene membranes. Thus, demonstrating a reliable performance over a long-term period is a prerequisite for graphene membranes to be promoted as a practical option in the gas separation market.

## Figures and Tables

**Table 1:** Properties of the selected gases [51,52].

Gas		Kinetic Diameter (Å)	Polarizability × 10^25^ (cm^3^)	Dipole Moment × 10^18^ (esu cm) ^(a)^	Quadrupole Moment × 10^26^ (esu cm^2^) ^(b)^
Helium	He ^(c)^	2.55	2.05	0	0
Water vapor	H_2_O ^(c)^	2.64	14.5	1.85	-
Hydrogen	H_2_	2.83	8.04	0	0.662
Carbon dioxide	CO_2_	3.30	29.1	0	4.30
Nitrogen	N_2_	3.64	17.4	0	1.52
Methane	CH_4_	3.80	25.9	0	0

^(a)^ Properties of polar molecules (presence of net force under uniform electric field). ^(b)^ Presence of net force under nonuniform electric field. ^(c)^ Helium (He) and water vapor (H_2_O) is included in the table as a reference.

**Table 2:** The syntheses of metal-organic framework/graphene oxide (MOF/GO) composites and their Brunauer-Emmett-Teller (BET) surface areas.

Composites ^(a)^	Particle Size of MOF (nm)	S_BET_ (m^2^/g)	% Increment in S_BET_^(b)^	Ref.
HKUST-1/1% rGO	-	1677	21.3	[70]
HKUST-1/16% GO	micron-sized	1550	3.3	[64]
HKUST-1/9% GO	10–40	1532	17.4	[68]
HKUST-1/9% GO	50	1002	10.2	[71]
HKUST-1/5% GO	-	1362	14.7	[72]
MIL-100/4% GO	-	1464	3.6	[73]
MOF-5/10% GO	50	806	1.6	[69]
MOF-5/5% B-GO	220–260	810	1.1	[74]
MOF-505/5% GO	micron-sized	1279	16.2	[75]
NiDOBDC/10% GO	20–35	1190	26.6	[76]
ZIF-8/1% GO	100–150	819	−26.9	[67]

^(a)^ The % indicated corresponds to weight percentage. ^(b)^ Percentage change is calculated with reference to the BET surface area of a pristine MOF. rGO: reduced graphene oxide.

**Table 3:** Performance of graphene-based membranes in H_2_/CO_2_ separation reported in the literature.

Membrane ^(a)^			Measurement Conditions	H_2_ Permeance (GPU)	H_2_/CO_2_ Selectivity	Year (Ref.)
Selective Layer	Thickness (nm)	Support				
GO (spin-casting)	5	PES5	- (pure gas, dry feed)	25	0.2	13′ [77]
GO (spin-casting)	5	PES5	- (pure gas, humidified feed)	12	0.1	13′ [77]
GO (spin-coating)	5	PES5	- (pure gas, dry feed)	35	35	13′ [77]
GO (spin-coating)	5	PES5	- (pure gas, humidified feed)	8.5	0.3	13′ [77]
GO	1.8	Al_2_O_3_	20 °C, H_2_/CO_2_ (1:1)	328	2500	13′ [78]
GO	9	Al_2_O_3_	20 °C, H_2_/CO_2_ (1:1)	343	3500	13′ [78]
GO	18	Al_2_O_3_	20 °C, H_2_/CO_2_ (1:1)	358	2000	13′ [78]
Graphene (1 layer)	0.345	PMMA	- (pure gas)	2.99 × 10^7^	4	14′ [79]
GO ^(b)^	-	Al_2_O_3_	H_2_/CO_2_ (1:1)	7	5.7	14′ [80]
ZIF-8@GO ^(b)^	-	Al_2_O_3_	H_2_/CO_2_ (1:1)	44	5.2	14′ [80]
ZIF-8@GO	100	Al_2_O_3_	250 °C, 1 bar, H_2_/CO_2_ (1:1)	433	14.9	14′ [80]
GO_M1_20	20,000	MCE	- (pure gas)	1.4	4.7	15′ [81]
GO_M3_20	20,000	MCE	- (pure gas)	2.4	3.5	15′ [81]
GO/ZIF-8	20	α-Al_2_O_3_	RT (pure gas)	280	633	16′ [82]
GO/ZIF-8	20	α-Al_2_O_3_	RT, H_2_/CO_2_ (1:1)	240	406	16′ [82]
GO (vacuum filtration)	-	Al_2_O_3_	RT, H_2_/CO_2_ (1:1)	1513	48	16′ [83]
GO (vacuum filtration)	-	Al_2_O_3_	RT (pure gas)	1746	58	16′ [83]
GO (spin-coating)	20	Al_2_O_3_	RT, H_2_/CO_2_ (1:1)	985	232	16′ [83]
GO (spin-coating)	20	Al_2_O_3_	RT (pure gas)	1045	259	16′ [83]
GO	890	Al_2_O_3_	2 bar, 25 °C (pure gas)	-	5	16′ [84]
EFDA-GO	890	Al_2_O_3_	2 bar, 25 °C (pure gas)	1326	28	16′ [84]
EFDA-GO	890	Al_2_O_3_	-, H_2_/CO_2_ (1:1)	876	17.2	16′ [84]
GO-0.5	1000	Al_2_O_3_	1 bar, 25 °C (pure gas)	81	20.9	17′ [85]
GO	230	YSZ	20 °C (pure gas)	133	111	17′ [86]
GO	2340	MCE	25 °C (pure gas)	2	2.0	17′ [87]
GOU (U: UiO-66-NH_2_)	1900	MCE	25 °C (pure gas)	116	6.4	17′ [87]
GO/U (U: UiO-66-NH_2_)	4100	MCE	25 °C (pure gas)	57	2.7	17′ [87]
GO (T-30) ^(c)^	320	α-Al_2_O_3_	RT (pure gas, dry feed)	400	15.0	17′ [88]
GO (T-30) ^(c)^	320	α-Al_2_O_3_	RT (pure gas, humidified feed)	313	10.5	17′ [88]
Graphene (1 layer)	0.345	AAO	- (pure gas)	4179	5.16	17′ [89]
GO	-	α-Al_2_O_3_	RT, H_2_/CO_2_ (1:1)	127	17.3	18′ [90]
GO ^(b)^	-	γ-Al_2_O_3_	3 bar, 20 °C (pure gas)	1761	38.5	18′ [91]
GO	-	Nylon	- (pure gas)	18,507	15	18′ [92]
CuO NS@GO-4	200	Nylon	- (pure gas)	22,687	18	18′ [92]
HKUST-1@GO-4	200	Nylon	- (pure gas)	4478	84	18′ [92]
HKUST-1@GO-4	200	Nylon	-, H_2_/CO_2_ (1:1)	1722	73.2	18′ [92]
GO-EDA-0	-	α-Al_2_O_3_	RT, H_2_/CO_2_ (1:1)	339	11.6	18′ [90]
GO-EDA-1	-	α-Al_2_O_3_	RT, H_2_/CO_2_ (1:1)	67	20.0	18′ [90]
GO-EDA-2	-	α-Al_2_O_3_	RT, H_2_/CO_2_ (1:1)	73	22.9	18′ [90]
GO-Zn_2_(bim)_4_-ZnO	200	α-Al_2_O_3_	1 bar (pure gas)	448	106	18′ [93]
GO-Zn_2_(bim)_4_-ZnO	200	α-Al_2_O_3_	1 bar. H_2_/CO_2_ (1:1)	448	89	18′ [93]
MEM-F200	500	PETE	RT (pure gas)	159	35.3	18′ [94]
MEM-L1	200	PETE	RT (pure gas)	397	35.3	18′ [95]
MEM-L1	200	PETE	RT, H_2_/CO_2_ (1:1)	340	22.5	18′ [95]
MEM-S1	-	PETE	RT (pure gas)	546	24.7	18′ [95]
MEM-S1	-	PETE	RT, H_2_/CO_2_ (1:1)	475	16.6	18′ [95]
MEM-S250	500	PETE	RT (pure gas)	169	26.4	18′ [94]
Graphene (1 layer) (M8)	0.345	Macroporous support	25 °C (pure gas)	17	7.4	18′ [96]
Graphene (1 layer) (M2)	0.345	Macroporous support	25 °C, H_2_/CO_2_ (1:1)	100	5.8	18′ [96]
GO ^(b)^	300	Al_2_O_3_	RT (pure gas)	134	44	19′ [97]
GO-SDBS ^(b)^	334	Al_2_O_3_	RT (pure gas)	239	337	19′ [97]
GO-B (B: Brodie)	200	Polyester	RT (pure gas)	141	80.7	19′ [98]
GO-H (H: Hummer)	200	Polyester	RT (pure gas)	399	35.3	19′ [98]
GO ^(b)^	470	Sil-1-Al_2_O_3_	1 bar, 25 °C (pure gas)	358	62	19′ [99]
Graphene (1 layer) (M9)	0.345	Macroporous W support	30 °C (pure gas)	1200	3	19′ [100]
Graphene (1 layer) (M9)	0.345	Macroporous W support	30 °C, H_2_/CO_2_ (1:1)	1200	3	19′ [100]
Graphene (1 layer)	0.345	Stainless steel mesh	- (pure gas)	1.55 × 10^8^	5.8	19′ [101]
Graphene (2 layer)	0.69	Stainless steel mesh	- (pure gas)	3.88 × 10^7^	6.1	19′ [101]
Graphene (4 layer)	1.38	Stainless steel mesh	- (pure gas)	2.09 × 10^7^	8.9	19′ [101]
GO	20,000	MCE	- (pure gas)	1800	4.2	20′ [102]
rGO_190_	20,000	MCE	- (pure gas)	2100	9.4	20′ [102]
GO	240	Nylon	1 bar, 25 °C (pure gas)	49	5.9	20′ [41]
GO-Co^2+^	240	Nylon	1 bar, 25 °C (pure gas)	60	6.4	20′ [41]
GO-La^3+^	240	Nylon	1 bar, 25 °C (pure gas)	90	8.7	20′ [41]
GO-500	41	Al_2_O_3_	1.5 bar, 25 °C (pure gas)	94	9.1	20′ [103]
CGO-76 (C: Cysteamine)	53	Al_2_O_3_	1.5 bar, 25 °C (pure gas)	52	21	20′ [103]
LCGO-40 (LC: L-cysteine)	-	Al_2_O_3_	1.5 bar, 25 °C (pure gas)	42	12	20′ [103]
GO	-	Nylon	1.2 bar, 25 °C, H_2_/CO_2_ (1:1)	11,600	9	20′ [104]
SOD/GO-M1	900	Nylon	1.2 bar, 25 °C, H_2_/CO_2_ (1:1)	1050	105	20′ [104]

^(a)^ All membrane configurations are indicated as flat sheets unless stated. ^(b)^ Self-supporting membrane. ^(c)^ Hollow fiber. GPU: gas permeation unit, RT: room temperature and PES5: polyethersulfone.

**Table 4:** Performance of graphene-based membranes in H_2_/N_2_ separation reported in the literature.

Membrane ^(a)^			Measurement Conditions	H_2_ Permeance (GPU)	H_2_/N_2_ Selectivity	Year (Ref.)
Skin Layer	Thickness (nm)	Support				
GO	1.8	Al_2_O_3_	20 °C, H_2_/N_2_ (1:1)	328	220	13′ [78]
GO	9	Al_2_O_3_	20 °C, H_2_/N_2_ (1:1)	343	300	13′ [78]
GO	18	Al_2_O_3_	20 °C, H_2_/N_2_ (1:1)	358	1000	13′ [78]
GO ^(b)^	-	Al_2_O_3_	H_2_/CO_2_ (1:1)	8	18.9	14′ [80]
ZIF-8@GO ^(b)^	-	Al_2_O_3_	H_2_/CO_2_ (1:1)	46	10.7	14′ [80]
ZIF-8@GO	100	Al_2_O_3_	250 °C, 1 bar, H_2_/CO_2_ (1:1)	433	90.5	14′ [80]
GO_M1_20	20,000	MCE	- (pure gas)	1.4	3.5	15′ [81]
GO_M3_20	20,000	MCE	- (pure gas)	2.4	6.9	15′ [81]
GO/ZIF-8	20	α-Al_2_O_3_	RT (pure gas)	280	88	16′ [82]
GO/ZIF-8	20	α-Al_2_O_3_	RT, H_2_/CO_2_ (1:1)	218	155	16′ [82]
GO (vacuum filtration)	-	Al_2_O_3_	RT (pure gas)	1746	65	16′ [83]
GO (spin coating)	20	Al_2_O_3_	RT (pure gas)	1045	292	16′ [83]
GO	890	Al_2_O_3_	0.2 MPa, 25 °C (pure gas)	-	3.0	16′ [84]
EFDA-GO	890	Al_2_O_3_	0.2 MPa, 25 °C (pure gas)	1123	7.5	16′ [84]
GO^[c]^	230	YSZ	20 °C (pure gas)	133	64	17′ [86]
GO	2340	MCE	25 °C (pure gas)	2	4.0	17′ [87]
GOU (U: UiO-66-NH_2_)	1900	MCE	25 °C (pure gas)	116	9.8	17′ [87]
GO/U (U: UiO-66-NH_2_)	4100	MCE	25 °C (pure gas)	57	7.0	17′ [87]
GO (T-30) ^(c)^	320	α-Al_2_O_3_	RT (pure gas, dry feed)	400	7.2	17′ [88]
GO (T-30) ^(c)^	320	α-Al_2_O_3_	RT (pure gas, humidified feed)	313	7.3	17′ [88]
GO	-	Nylon	- (pure gas)	18,507	18	18′ [92]
CuO NS@GO-4	200	Nylon	- (pure gas)	22,687	21	18′ [92]
HKUST-1@GO-4	200	Nylon	- (pure gas)	4478	54	18′ [92]
GO ^(b)^	-	γ-Al_2_O_3_	3 bar, 20 °C (pure gas)	1761	16.5	18′ [91]
GO-Zn_2_(bim)_4_-ZnO	200	α-Al_2_O_3_	1 bar (pure gas)	448	126	18′ [93]
GO-Zn_2_(bim)_4_-ZnO	200	α-Al_2_O_3_	1 bar. H_2_/N_2_ (1:1)	448	103	18′ [93]
MEM-F200	500	PETE	RT (pure gas)	159	31.5	18′ [94]
MEM-L1	200	PETE	RT (pure gas)	397	29.6	18′ [95]
MEM-S1	-	PETE	RT (pure gas)	546	18.3	18′ [95]
MEM-S250	500	PETE	RT (pure gas)	169	23.7	18′ [94]
GO ^(b)^	300	Al_2_O_3_	RT (pure gas)	134	36	19′ [97]
GO ^(b)^	470	Sil-1-Al_2_O_3_	1 bar, 25 °C (pure gas)	358	50	19′ [99]
GO ^(b)^	470	Sil-1-Al_2_O_3_	1 bar, 20 °C, H_2_/N_2_ (1:1)	328	40.7	19′ [99]
GO-SDBS ^(b)^	334	Al_2_O_3_	RT (pure gas)	239	44	19′ [97]
GO-B	200	Polyester	RT (pure gas)	141	60.1	19′ [98]
GO-H	200	Polyester	RT (pure gas)	399	31.5	19′ [98]

^(a)^ All membrane configurations are indicated as flat sheets unless stated. ^(b)^ Self-supporting membrane. ^(c)^ Hollow fiber.

**Table 5:** Performance of graphene-based membranes in H_2_/CH_4_ separation reported in the literature.

Membrane ^(a)^			Measurement Conditions	H_2_ Permeance (GPU)	H_2_/CH_4_ Selectivity	Year (Ref.)
Skin Layer	Thickness (nm)	Support				
GO ^(b)^	-	Al_2_O_3_	H_2_/CH_4_ (1:1)	8	38.4	14′ [80]
ZIF-8@GO ^(b)^	-	Al_2_O_3_	H_2_/CH_4_ (1:1)	44	12.8	14′ [80]
ZIF-8@GO	100	Al_2_O_3_	250 °C, 1 bar, H_2_/CH_4_ (1:1)	433	139.1	14′ [80]
GO_M3_20	20,000	MCE	- (pure gas)	2.4	5.6	15′ [81]
GO/ZIF-8	20	α-Al_2_O_3_	RT (pure gas)	280	162	16′ [82]
GO/ZIF-8	20	α-Al_2_O_3_	RT, H_2_/CH_4_ (1:1)	218	355	16′ [82]
GO (vacuum filtration)	-	Al_2_O_3_	RT (pure gas)	1746	29.3	16′ [83]
GO (spin coating)	20	Al_2_O_3_	RT (pure gas)	1045	194	16′ [83]
GO (8 wt%)/PSF **^(c)^**	50,000	-	35 °C (pure gas)	0.1	28	17′ [105]
GO(30)_UiO-66_48h (8 wt%)/PSF **^(c)^**	50,000	-	35 °C (pure gas)	0.32	68	17′ [105]
GO (8 wt%)/PI **^(c)^**	50,000	-	35 °C (pure gas)	0.26	81	17′ [105]
GO(30)_UiO-66_48h (8 wt%)/PI **^(c)^**	50,000	-	35 °C (pure gas)	0.94	140	17′ [105]
GO	230	YSZ	20 °C (pure gas)	133	53	17′ [86]
GO (T-30) ^(d)^	320	α-Al_2_O_3_	RT (pure gas, dry feed)	400	6.4	17′ [88]
GO (T-30) ^(d)^	320	α-Al_2_O_3_	RT (pure gas, humidified feed)	313	6.5	17′ [88]
Graphene (1 layer)	0.345	AAO	- (pure gas)	4179	3.17	17′ [89]
GO	-	Nylon	- (pure gas)	18,507	3	18′ [92]
CuO NS@GO-4	200	Nylon	- (pure gas)	22,687	4	18′ [92]
HKUST-1@GO-4	200	Nylon	- (pure gas)	4478	35	18′ [92]
GO-Zn_2_(bim)_4_-ZnO	200	α-Al_2_O_3_	1 bar (pure gas)	448	256	18′ [93]
GO-Zn_2_(bim)_4_-ZnO	200	α-Al_2_O_3_	1 bar. H_2_/CH_4_ (1:1)	448	221	18′ [93]
MEM-L1	200	PETE	RT (pure gas)	397	16.6	18′ [95]
MEM-S1	-	PETE	RT (pure gas)	546	11.4	18′ [95]
MEM-F200	500	PETE	RT (pure gas)	159	15.7	18′ [94]
MEM-S250	500	PETE	RT (pure gas)	169	12.5	18′ [94]
Graphene (1 layer) (M5)	0.345	Macroporous support	25 °C (pure gas)	210	12.8	18′ [96]
Graphene (1 layer) (M2)	0.345	Macroporous support	25 °C, H_2_/CH_4_ (1:1)	100	11	18′ [96]
GO ^(c)^	300	Al_2_O_3_	RT (pure gas)	134	14.5	19′ [97]
GO-SDBS	334	Al_2_O_3_	RT (pure gas)	239	20.5	19′ [97]
GO ^(c)^	470	Sil-1-Al_2_O_3_	1 bar, 25 °C (pure gas)	358	28	19′ [99]
GO-B	200	Polyester	RT (pure gas)	141	27.7	19′ [98]
GO-H	200	Polyester	RT (pure gas)	399	15.7	19′ [98]
Graphene (1 layer) (M4)	0.345	Macroporous W support	30 °C (pure gas)	1000	5.1	19′ [106]
Graphene (1 layer) (M9)	0.345	Macroporous W support	30 °C (pure gas)	1200	16	19′ [100]
Graphene (1 layer) (M9)	0.345	Macroporous W support	30 °C, H_2_/CH_4_ (1:1)	1200	16	19′ [100]
Graphene (1 layer)	0.345	Stainless steel mesh	- (pure gas)	1.55 × 10^8^	2.5	19′ [101]
Graphene (2 layers)	0.69	Stainless steel mesh	- (pure gas)	3.88 × 10^7^	2.3	19′ [101]
Graphene (4 layers)	1.38	Stainless steel mesh	- (pure gas)	2.09 × 10^7^	3.0	19′ [101]
Graphene (1 layer)	0.345	Nanoporous carbon (NPC)	- (pure gas)	1090	9.5	20′ [107]

^(a)^ The membrane configurations are flat sheets unless stated. ^(b)^ Self-supporting membrane. ^(c)^ The values are reported in barrer (dense flat sheet membrane), where 1 barrer = 10^−10^ cm^3^ (STP) cm cm^−2^ s^−1^ cmHg^−1^. Nevertheless, the values in the table are converted to GPU for an effective comparison. ^(d)^ Hollow fiber.

**Table 6:** Summary of the upper bounds [28,29,35,129] used for H_2_ separation ^(a)^.

Upper Bound Curve	Knudsen Selectivity	1991		2008		2015 (2018) ^(b)^	
		*k (barrer)*	*n*	*k (barrer)*	*n*	*k (barrer)*	*n*
H_2_/CO_2_	4.69	1200	−1.94	4515	−2.30	15,248 ^(c)^	−1.89 ^(c)^
H_2_/N_2_	3.74	52,918	−1.53	97,650	−1.48	1,100,000	−1.46
H_2_/CH_4_	2.83	18,500	−1.21	27,200	−1.11	195,000	−1.10

^(a)^ The upper-bound curve can be constructed using $P=k\alpha^{n}$, where *P* = permeability, *α* = selectivity and *k* and *n* are constants. ^(b)^ The latest H_2_/N_2_ and H_2_/CH_4_ upper-bound limits were constructed in 2015, whereas the latest H_2_/CO_2_ upper-bound limit was constructed in 2018. ^(c)^ The parameters (*k* and *n*) in the 2018 upper-bound limit for H_2_/CO_2_ separation were not furnished. The limit is determined based on interpolation of the available data points.

## Nomenclature

Symbol	Full Name
Al_2_O_3_	Alumina (aluminum oxide)
BET	Brunauer-Emmett-Teller
COF	Covalent organic framework
Cu	Copper
CVD	Chemical vapor deposition
DFT	Density functional theory
EDA	Ethylenediamine
EFDA	External force driven assembly
FIB	Focused ion beam
FTIR	Fourier transform infrared spectroscopy
GO	Graphene oxide
GPU	Gas permeation unit
ISA	Integrally skin asymmetric
LDA	Local density approximation
MCE	Mixed cellulose ester
MD	Molecular dynamics
MMM	Mixed-matrix membrane
MOF	Metal-organic framework
MOP	Microporous organic polymer
NPC	Nanoporous carbon
NS	Nanosheet
ODPA	4,4′-oxydiphthalic anhydride
PBE	Perdew, Burke and Erzenhoff
PES5	Polyethersulfone
PETE	Polyester track etch
PI	Polyimide (Matrimid^®^ 5218)
PMMA	Poly(methyl methacrylate)
PSA	Pressure swing adsorption
PSF	Polysulfone
rGO	Reduced graphene oxide
RT	Room temperature
SDBS	Sodium dodecylbenzenesulfonate
Sil-1-Al_2_O_3_	Silicalite-1 modified alumina
SOD	Hydroxy sodalite
STEM	Scanning transmission electron microscopy
TMPDA	2,4,6-trimethyl-*m*-phenylenediamine
vdW-DF	van der Waals density functional
YSZ	Yttrium-stabilized zirconia (ceramic)

## References

[1] Ahmadpour J., Ahmadi M., Javdani A. (2019). Hydrodesulfurization unit for natural gas condensate. J. Therm. Anal. Calorim..

[2] Kadijani J.A., Narimani E. (2016). Simulation of hydrodesulfurization unit for natural gas condensate with high sulfur content. Appl. Petrochem. Res..

[3] Frauzem R., Kongpanna P., Roh K., Lee J.H., Pavarajarn V., Assabumrungrat S., Gani R., You F. (2015). Chapter 7—Sustainable Process Design: Sustainable Process Networks for Carbon Dioxide Conversion. Computer Aided Chemical Engineering.

[4] Germeshuizen L.M., Blom P. (2013). A techno-economic evaluation of the use of hydrogen in a steel production process, utilizing nuclear process heat. Int. J. Hydrog. Energy.

[5] Grundt T., Christiansen K. (1982). Hydrogen by water electrolysis as basis for small scale ammonia production. A comparison with hydrocarbon based technologies. Int. J. Hydrog. Energy.

[6] Yang E., Alayande A.B., Goh K., Kim C.-M., Chu K.-H., Hwang M.-H., Ahn J.-H., Chae K.-J. (2020). 2D materials-based membranes for hydrogen purification: Current status and future prospects. Int. J. Hydrog. Energy.

[7] Chuah C.Y., Goh K., Yang Y., Gong H., Li W., Karahan H.E., Guiver M.D., Wang R., Bae T.-H. (2018). Harnessing filler materials for enhancing biogas separation membranes. Chem. Rev..

[8] Chuah C.Y., Kim K., Lee J., Koh D.-Y., Bae T.-H. (2019). CO_2_ absorption using membrane contactors: Recent progress and future perspective. Ind. Eng. Chem. Res..

[9] IEA The Future of Hydrogen. https://www.iea.org/reports/the-future-of-hydrogen.

[10] OFFICE of ENERGY EFFICIENCY and RENEWABLE ENERGY Hydrogen Production: Natural Gas Reforming. https://www.energy.gov/eere/fuelcells/hydrogen-production-natural-gas-reforming.

[11] Vozniuk O., Tanchoux N., Millet J.-M., Albonetti S., Di Renzo F., Cavani F., Albonetti S., Perathoner S., Quadrelli E.A. (2019). Chapter 14—Spinel Mixed Oxides for Chemical-Loop Reforming: From Solid State to Potential Application. Studies in Surface Science and Catalysis.

[12] Navarro Yerga R.M., Alvarez-Galván M.C., Vaquero F., Arenales J., Fierro J.L.G., Gandía L.M., Arzamendi G., Diéguez P.M. (2013). Chapter 3—Hydrogen Production from Water Splitting Using Photo-Semiconductor Catalysts. Renewable Hydrogen Technologies.

[13] Chuah C.Y., Goh K., Bae T.-H. (2017). Hierarchically structured HKUST-1 nanocrystals for enhanced SF_6_ capture and recovery. J. Phys. Chem. C.

[14] Chuah C.Y., Yu S., Na K., Bae T.-H. (2018). Enhanced SF_6_ recovery by hierarchically structured MFI zeolite. J. Ind. Eng. Chem..

[15] Chuah C.Y., Yang Y., Bae T.-H. (2018). Hierarchically porous polymers containing triphenylamine for enhanced SF_6_ separation. Micropor. Mesopor. Mater..

[16] Yang Y., Goh K., Chuah C.Y., Karahan H.E., Birer Ö., Bae T.-H. (2019). Sub-Ångström-level engineering of ultramicroporous carbons for enhanced sulfur hexafluoride capture. Carbon.

[17] Tao W., Ma S., Xiao J., Bénard P., Chahine R. (2019). Simulation and optimization for hydrogen purification performance of vacuum pressure swing adsorption. Energy Procedia.

[18] Xu G., Liang F., Yang Y., Hu Y., Zhang K., Liu W. (2014). An Improved CO_2_ Separation and Purification System Based on Cryogenic Separation and Distillation Theory. Energies.

[19] Wongchitphimon S., Lee S.S., Chuah C.Y., Wang R., Bae T.H. (2020). Composite Materials for Carbon Capture.

[20] Zhang X., Chuah C.Y., Dong P., Cha Y.-H., Bae T.-H., Song M.-K. (2018). Hierarchically porous Co-MOF-74 hollow nanorods for enhanced dynamic CO_2_ separation. ACS Appl. Mater. Interfaces.

[21] Lee J., Chuah C.Y., Kim J., Kim Y., Ko N., Seo Y., Kim K., Bae T.H., Lee E. (2018). Separation of Acetylene from Carbon Dioxide and Ethylene by a Water-Stable Microporous Metal–Organic Framework with Aligned Imidazolium Groups inside the Channels. Angew. Chem. Int. Ed..

[22] Samarasinghe S.A.S.C., Chuah C.Y., Li W., Sethunga G.S.M.D.P., Wang R., Bae T.-H. (2019). Incorporation of Co^III^ acetylacetonate and SNW-1 nanoparticles to tailor O_2_/N_2_ separation performance of mixed-matrix membrane. Sep. Purif. Technol..

[23] Chuah C.Y., Samarasinghe S.A.S.C., Li W., Goh K., Bae T.-H. (2020). Leveraging Nanocrystal HKUST-1 in Mixed-Matrix Membranes for Ethylene/Ethane Separation. Membranes.

[24] Samarasinghe S.A.S.C., Chuah C.Y., Karahan H.E., Sethunga G.S.M.D.P., Bae T.H. (2020). Enhanced O_2_/N_2_ Separation of Mixed-Matrix Membrane Filled with Pluronic-Compatibilized Cobalt Phthalocyanine Particles. Membranes.

[25] Hinchliffe A.B., Porter K.E. (2000). A Comparison of Membrane Separation and Distillation. Chem. Eng. Res. Des..

[26] Gong H., Chuah C.Y., Yang Y., Bae T.-H. (2018). High performance composite membranes comprising Zn(pyrz)_2_(SiF_6_) nanocrystals for CO_2_/CH_4_ separation. J. Ind. Eng. Chem..

[27] Wongchitphimon S., Rongwong W., Chuah C.Y., Wang R., Bae T.-H. (2017). Polymer-fluorinated silica composite hollow fiber membranes for the recovery of biogas dissolved in anaerobic effluent. J. Membr. Sci..

[28] Robeson L.M. (1991). Correlation of separation factor versus permeability for polymeric membranes. J. Membr. Sci..

[29] Robeson L.M. (2008). The upper bound revisited. J. Membr. Sci..

[30] Chuah C.Y., Bae T.-H. (2019). Incorporation of Cu_3_BTC_2_ nanocrystals to increase the permeability of polymeric membranes in O_2_/N_2_ separation. BMC Chem. Eng..

[31] Pandey P., Chauhan R.S. (2001). Membranes for gas separation. Prog. Polym. Sci..

[32] Chuah C.Y., Lee J., Song J., Bae T.-H. (2020). CO_2_/N_2_ Separation Properties of Polyimide-Based Mixed-Matrix Membranes Comprising UiO-66 with Various Functionalities. Membranes.

[33] Pera-Titus M. (2014). Porous Inorganic Membranes for CO_2_ Capture: Present and Prospects. Chem. Rev..

[34] Li W., Goh K., Chuah C.Y., Bae T.-H. (2019). Mixed-matrix carbon molecular sieve membranes using hierarchical zeolite: A simple approach towards high CO_2_ permeability enhancements. J. Membr. Sci..

[35] Ding L., Wei Y., Li L., Zhang T., Wang H., Xue J., Ding L.-X., Wang S., Caro J., Gogotsi Y. (2018). MXene molecular sieving membranes for highly efficient gas separation. Nat. Commun..

[36] Karahan H.E., Goh K., Zhang C., Yang E., Yıldırım C., Chuah C.Y., Ahunbay M.G., Lee J., Tantekin-Ersolmaz Ş.B., Chen Y. (2020). MXene Materials for Designing Advanced Separation Membranes. Adv. Mater..

[37] Wang D., Wang Z., Wang L., Hu L., Jin J. (2015). Ultrathin membranes of single-layered MoS_2_ nanosheets for high-permeance hydrogen separation. Nanoscale.

[38] Liu Y., Wang N., Caro J. (2014). In situ formation of LDH membranes of different microstructures with molecular sieve gas selectivity. J. Mater. Chem. A.

[39] Peng Y., Li Y., Ban Y., Jin H., Jiao W., Liu X., Yang W. (2014). Metal-organic framework nanosheets as building blocks for molecular sieving membranes. Science.

[40] Fan H., Mundstock A., Feldhoff A., Knebel A., Gu J., Meng H., Caro J. (2018). Covalent Organic Framework–Covalent Organic Framework Bilayer Membranes for Highly Selective Gas Separation. J. Am. Chem. Soc..

[41] Chuah C.Y., Nie L., Lee J.-M., Bae T.-H. (2020). The influence of cations intercalated in graphene-oxide membranes in tuning H_2_/CO_2_ separation performance. Sep. Purif. Technol..

[42] Zhang Y., Shi Q., Liu Y., Wang Y., Meng Z., Xiao C., Deng K., Rao D., Lu R. (2015). Hexagonal Boron Nitride with Designed Nanopores as a High-Efficiency Membrane for Separating Gaseous Hydrogen from Methane. J. Phys. Chem. C.

[43] Huang K., Liu G., Lou Y., Dong Z., Shen J., Jin W. (2014). A Graphene Oxide Membrane with Highly Selective Molecular Separation of Aqueous Organic Solution. Angew. Chem. Int. Ed..

[44] Hu Y., Dong X., Nan J., Jin W., Ren X., Xu N., Lee Y.M. (2011). Metal–organic framework membranes fabricated via reactive seeding. Chem. Commun..

[45] Li W., Su P., Li Z., Xu Z., Wang F., Ou H., Zhang J., Zhang G., Zeng E. (2017). Ultrathin metal–organic framework membrane production by gel–vapour deposition. Nat. Commun..

[46] Nie L., Chuah C.Y., Bae T.H., Lee J.M. (2020). Graphene-Based Advanced Membrane Applications in Organic Solvent Nanofiltration. Adv. Func. Mater..

[47] Li W., Chuah C.Y., Nie L., Bae T.-H. (2019). Enhanced CO_2_/CH_4_ selectivity and mechanical strength of mixed-matrix membrane incorporated with NiDOBDC/GO composite. J. Ind. Eng. Chem..

[48] Norahim N., Faungnawakij K., Quitain A.T., Klaysom C. (2019). Composite membranes of graphene oxide for CO_2_/CH_4_ separation. J. Chem. Tech. Biotechnol..

[49] Ali A., Pothu R., Siyal S.H., Phulpoto S., Sajjad M., Thebo K.H. (2019). Graphene-based membranes for CO_2_ separation. Mater. Sci. Energy Technol..

[50] Alen S.K., Nam S., Dastgheib S.A. (2019). Recent advances in graphene oxide membranes for gas separation applications. Int. J. Mol. Sci..

[51] Li J.-R., Kuppler R.J., Zhou H.-C. (2009). Selective gas adsorption and separation in metal–organic frameworks. Chem. Soc. Rev..

[52] Chuah C.Y. (2019). Microporous Materials with Tailored Structural Properties for Enhanced Gas Separation.

[53] Liu G., Jin W., Xu N. (2015). Graphene-based membranes. Chem. Soc. Rev..

[54] Goh K., Karahan H.E., Yang E., Bae T.-H. (2019). Graphene-Based membranes for CO_2_/CH_4_ separation: Key challenges and perspectives. Appl. Sci..

[55] Goh K., Karahan H.E., Wei L., Bae T.-H., Fane A.G., Wang R., Chen Y. (2016). Carbon nanomaterials for advancing separation membranes: A strategic perspective. Carbon.

[56] Bunch J.S., Verbridge S.S., Alden J.S., Van Der Zande A.M., Parpia J.M., Craighead H.G., McEuen P.L. (2008). Impermeable atomic membranes from graphene sheets. Nano Lett..

[57] Yoo B.M., Shin H.J., Yoon H.W., Park H.B. (2014). Graphene and graphene oxide and their uses in barrier polymers. J. Appl. Polym. Sci..

[58] Berry V. (2013). Impermeability of graphene and its applications. Carbon.

[59] Leenaerts O., Partoens B., Peeters F. (2008). Graphene: A perfect nanoballoon. Appl. Phys. Lett..

[60] Wilkes J.O., Birmingham S.G. (2006). Fluid Mechanics for Chemical Engineers with Microfluidics and CFD.

[61] Nielsen L.E. (1967). Models for the permeability of filled polymer systems. J. Macromol. Sci. Chem..

[62] Yang E., Goh K., Chuah C.Y., Wang R., Bae T.-H. (2020). Asymmetric mixed-matrix membranes incorporated with nitrogen-doped graphene nanosheets for highly selective gas separation. J. Membr. Sci..

[63] Yang E., Karahan H.E., Goh K., Chuah C.Y., Wang R., Bae T.-H. (2019). Scalable fabrication of graphene-based laminate membranes for liquid and gas separations by crosslinking-induced gelation and doctor-blade casting. Carbon.

[64] Li W., Chuah C.Y., Yang Y., Bae T.-H. (2018). Nanocomposites formed by in situ growth of NiDOBDC nanoparticles on graphene oxide sheets for enhanced CO_2_ and H_2_ storage. Micropor. Mesopor. Mater..

[65] Li W., Samarasinghe S.A.S.C., Bae T.-H. (2018). Enhancing CO_2_/CH_4_ separation performance and mechanical strength of mixed-matrix membrane via combined use of graphene oxide and ZIF-8. J. Ind. Eng. Chem..

[66] Brunauer S., Emmett P.H., Teller E. (1938). Adsorption of Gases in Multimolecular Layers. J. Am. Chem. Soc..

[67] Kumar R., Jayaramulu K., Maji T.K., Rao C. (2013). Hybrid nanocomposites of ZIF-8 with graphene oxide exhibiting tunable morphology, significant CO_2_ uptake and other novel properties. Chem. Commun..

[68] Liu S., Sun L., Xu F., Zhang J., Jiao C., Li F., Li Z., Wang S., Wang Z., Jiang X. (2013). Nanosized Cu-MOFs induced by graphene oxide and enhanced gas storage capacity. Energy Environ. Sci..

[69] Petit C., Bandosz T.J. (2009). MOF–graphite oxide composites: Combining the uniqueness of graphene layers and metal–organic frameworks. Adv. Mater..

[70] Huang W., Zhou X., Xia Q., Peng J., Wang H., Li Z. (2014). Preparation and adsorption performance of GrO@ Cu-BTC for separation of CO_2_/CH_4_. Ind. Eng. Chem. Res..

[71] Petit C., Burress J., Bandosz T.J. (2011). The synthesis and characterization of copper-based metal–organic framework/graphite oxide composites. Carbon.

[72] Li Y., Miao J., Sun X., Xiao J., Li Y., Wang H., Xia Q., Li Z. (2016). Mechanochemical synthesis of Cu-BTC@ GO with enhanced water stability and toluene adsorption capacity. Chem. Eng. J..

[73] Petit C., Bandosz T.J. (2011). Synthesis, characterization, and ammonia adsorption properties of mesoporous metal–organic framework (MIL (Fe))–graphite oxide composites: Exploring the limits of materials fabrication. Adv. Func. Mater..

[74] Jahan M., Bao Q., Yang J.-X., Loh K.P. (2010). Structure-directing role of graphene in the synthesis of metal− organic framework nanowire. J. Am. Chem. Soc..

[75] Chen Y., Lv D., Wu J., Xiao J., Xi H., Xia Q., Li Z. (2017). A new MOF-505@ GO composite with high selectivity for CO_2_/CH_4_ and CO_2_/N_2_ separation. Chem. Eng. J..

[76] Andrea D., Szilvia K., János M., György S., Ying W., Krisztina L. (2020). Graphene Oxide Protected Copper Benzene-1,3,5-Tricarboxylate for Clean Energy Gas Adsorption. Nanomaterials.

[77] Kim H.W., Yoon H.W., Yoon S.-M., Yoo B.M., Ahn B.K., Cho Y.H., Shin H.J., Yang H., Paik U., Kwon S. (2013). Selective gas transport through few-layered graphene and graphene oxide membranes. Science.

[78] Li H., Song Z., Zhang X., Huang Y., Li S., Mao Y., Ploehn H.J., Bao Y., Yu M. (2013). Ultrathin, molecular-sieving graphene oxide membranes for selective hydrogen separation. Science.

[79] Celebi K., Buchheim J., Wyss R.M., Droudian A., Gasser P., Shorubalko I., Kye J.-I., Lee C., Park H.G. (2014). Ultimate Permeation Across Atomically Thin Porous Graphene. Science.

[80] Huang A., Liu Q., Wang N., Zhu Y., Caro J.r. (2014). Bicontinuous zeolitic imidazolate framework ZIF-8@ GO membrane with enhanced hydrogen selectivity. J. Am. Chem. Soc..

[81] Romanos G., Pastrana-Martínez L., Tsoufis T., Athanasekou C., Galata E., Katsaros F., Favvas E., Beltsios K., Siranidi E., Falaras P. (2015). A facile approach for the development of fine-tuned self-standing graphene oxide membranes and their gas and vapor separation performance. J. Membr. Sci..

[82] Wang X., Chi C., Tao J., Peng Y., Ying S., Qian Y., Dong J., Hu Z., Gu Y., Zhao D. (2016). Improving the hydrogen selectivity of graphene oxide membranes by reducing non-selective pores with intergrown ZIF-8 crystals. Chem. Commun..

[83] Chi C., Wang X., Peng Y., Qian Y., Hu Z., Dong J., Zhao D. (2016). Facile preparation of graphene oxide membranes for gas separation. Chem. Mater..

[84] Shen J., Liu G., Huang K., Chu Z., Jin W., Xu N. (2016). Subnanometer two-dimensional graphene oxide channels for ultrafast gas sieving. ACS Nano.

[85] Guan K., Shen J., Liu G., Zhao J., Zhou H., Jin W. (2017). Spray-evaporation assembled graphene oxide membranes for selective hydrogen transport. Sep. Purif. Technol..

[86] Zhu J., Meng X., Zhao J., Jin Y., Yang N., Zhang S., Sunarso J., Liu S. (2017). Facile hydrogen/nitrogen separation through graphene oxide membranes supported on YSZ ceramic hollow fibers. J. Membr. Sci..

[87] Jia M., Feng Y., Liu S., Qiu J., Yao J. (2017). Graphene oxide gas separation membranes intercalated by UiO-66-NH_2_ with enhanced hydrogen separation performance. J. Membr. Sci..

[88] Huang K., Yuan J., Shen G., Liu G., Jin W. (2017). Graphene oxide membranes supported on the ceramic hollow fibre for efficient H_2_ recovery. Chin. J. Chem. Eng..

[89] Boutilier M.S.H., Jang D., Idrobo J.-C., Kidambi P.R., Hadjiconstantinou N.G., Karnik R. (2017). Molecular Sieving Across Centimeter-Scale Single-Layer Nanoporous Graphene Membranes. ACS Nano.

[90] Lin H., Liu R., Dangwal S., Kim S.-J., Mehra N., Li Y., Zhu J. (2018). Permselective H_2_/CO_2_ separation and desalination of hybrid GO/rGO membranes with controlled pre-cross-linking. ACS Appl. Mater. Interfaces.

[91] Zeynali R., Ghasemzadeh K., Sarand A.B., Kheiri F., Basile A. (2018). Performance evaluation of graphene oxide (GO) nanocomposite membrane for hydrogen separation: Effect of dip coating sol concentration. Sep. Purif. Technol..

[92] Kang Z., Wang S., Fan L., Zhang M., Kang W., Pang J., Du X., Guo H., Wang R., Sun D. (2018). In situ generation of intercalated membranes for efficient gas separation. Commun. Chem..

[93] Li Y., Liu H., Wang H., Qiu J., Zhang X. (2018). GO-guided direct growth of highly oriented metal–organic framework nanosheet membranes for H_2_/CO_2_ separation. Chem. Sci..

[94] Ibrahim A.F., Lin Y. (2018). Synthesis of graphene oxide membranes on polyester substrate by spray coating for gas separation. Chem. Eng. Sci..

[95] Ibrahim A., Lin Y. (2018). Gas permeation and separation properties of large-sheet stacked graphene oxide membranes. J. Membr. Sci..

[96] Huang S., Dakhchoune M., Luo W., Oveisi E., He G., Rezaei M., Zhao J., Alexander D.T.L., Züttel A., Strano M.S. (2018). Single-layer graphene membranes by crack-free transfer for gas mixture separation. Nat. Commun..

[97] Ma S., Tang Z., Fan Y., Zhao J., Meng X., Yang N., Zhuo S., Liu S. (2019). Surfactant-modified graphene oxide membranes with tunable structure for gas separation. Carbon.

[98] Ibrahim A.F., Banihashemi F., Lin Y. (2019). Graphene oxide membranes with narrow inter-sheet galleries for enhanced hydrogen separation. Chem. Commun..

[99] Meng X., Fan Y., Zhu J., Jin Y., Li C., Yang N., Zhao J., Sunarso J., Liu S. (2019). Improving hydrogen permeation and interface property of ceramic-supported graphene oxide membrane via embedding of silicalite-1 zeolite into Al_2_O_3_ hollow fiber. Sep. Purif. Technol..

[100] Zhao J., He G., Huang S., Villalobos L.F., Dakhchoune M., Bassas H., Agrawal K.V. (2019). Etching gas-sieving nanopores in single-layer graphene with an angstrom precision for high-performance gas mixture separation. Sci. Adv..

[101] Shimizu K., Ohba T. (2017). Extremely permeable porous graphene with high H_2_/CO_2_ separation ability achieved by graphene surface rejection. Phys. Chem. Chem. Phys..

[102] Lee S.E., Jang J., Kim J., Woo J.Y., Seo S., Jo S., Kim J.-W., Jeon E.-S., Jung Y., Han C.-S. (2020). Tunable sieving of small gas molecules using horizontal graphene oxide membrane. J. Membr. Sci..

[103] Cheng L., Guan K., Liu G., Jin W. (2020). Cysteamine-crosslinked graphene oxide membrane with enhanced hydrogen separation property. J. Membr. Sci..

[104] Guo H., Kong G., Yang G., Pang J., Kang Z., Feng S., Zhao L., Fan L., Zhu L., Vicente A. (2020). Cross-Linking between Sodalite Nanoparticles and Graphene Oxide in Composite Membranes to Trigger High Gas Permeance, Selectivity, and Stability in Hydrogen Separation. Angew. Chem. Int. Ed..

[105] Castarlenas S., Téllez C., Coronas J. (2017). Gas separation with mixed matrix membranes obtained from MOF UiO-66-graphite oxide hybrids. J. Membr. Sci..

[106] Khan M.H., Moradi M., Dakhchoune M., Rezaei M., Huang S., Zhao J., Agrawal K.V. (2019). Hydrogen sieving from intrinsic defects of benzene-derived single-layer graphene. Carbon.

[107] Rezaei M., Li S., Huang S., Agrawal K.V. (2020). Hydrogen-sieving single-layer graphene membranes obtained by crystallographic and morphological optimization of catalytic copper foil. J. Membr. Sci..

[108] Koenig S.P., Wang L., Pellegrino J., Bunch J.S. (2012). Selective molecular sieving through porous graphene. Nat. Nanotechnol..

[109] Stankovich S., Piner R.D., Chen X., Wu N., Nguyen S.T., Ruoff R.S. (2006). Stable aqueous dispersions of graphitic nanoplatelets via the reduction of exfoliated graphite oxide in the presence of poly (sodium 4-styrenesulfonate). J. Mater. Chem..

[110] Dikin D.A., Stankovich S., Zimney E.J., Piner R.D., Dommett G.H., Evmenenko G., Nguyen S.T., Ruoff R.S. (2007). Preparation and characterization of graphene oxide paper. Nature.

[111] Tsou C.-H., An Q.-F., Lo S.-C., De Guzman M., Hung W.-S., Hu C.-C., Lee K.-R., Lai J.-Y. (2015). Effect of microstructure of graphene oxide fabricated through different self-assembly techniques on 1-butanol dehydration. J. Membr. Sci..

[112] Chen L., Shi G., Shen J., Peng B., Zhang B., Wang Y., Bian F., Wang J., Li D., Qian Z. (2017). Ion sieving in graphene oxide membranes via cationic control of interlayer spacing. Nature.

[113] Stankovich S., Dikin D.A., Compton O.C., Dommett G.H., Ruoff R.S., Nguyen S.T. (2010). Systematic post-assembly modification of graphene oxide paper with primary alkylamines. Chem. Mater..

[114] Gao Y., Liu L.-Q., Zu S.-Z., Peng K., Zhou D., Han B.-H., Zhang Z. (2011). The effect of interlayer adhesion on the mechanical behaviors of macroscopic graphene oxide papers. ACS Nano.

[115] Hummers Jr W.S., Offeman R.E. (1958). Preparation of graphitic oxide. J. Am. Chem. Soc..

[116] Brodie B.C. (1859). XIII. On the atomic weight of graphite. Philosophical Transactions of the Royal Society of London.

[117] Samarasinghe S., Chuah C.Y., Yang Y., Bae T.-H. (2018). Tailoring CO_2_/CH_4_ separation properties of mixed-matrix membranes via combined use of two-and three-dimensional metal-organic frameworks. J. Membr. Sci..

[118] Chuah C.Y., Li W., Samarasinghe S., Sethunga G., Bae T.-H. (2019). Enhancing the CO_2_ separation performance of polymer membranes via the incorporation of amine-functionalized HKUST-1 nanocrystals. Micropor. Mesopor. Mater..

[119] Schrier J. (2010). Helium separation using porous graphene membranes. J. Phys. Chem. Lett..

[120] Jiang D.-e., Cooper V.R., Dai S. (2009). Porous graphene as the ultimate membrane for gas separation. Nano Lett..

[121] Du H., Li J., Zhang J., Su G., Li X., Zhao Y. (2011). Separation of hydrogen and nitrogen gases with porous graphene membrane. J. Phys. Chem. C.

[122] Drahushuk L.W., Strano M.S. (2012). Mechanisms of gas permeation through single layer graphene membranes. Langmuir.

[123] Tao Y., Xue Q., Liu Z., Shan M., Ling C., Wu T., Li X. (2014). Tunable hydrogen separation in porous graphene membrane: First-principle and molecular dynamic simulation. ACS Appl. Mater. Interfaces.

[124] Zheng H., Zhu L., He D., Guo T., Li X., Chang X., Xue Q. (2017). Two-dimensional graphene oxide membrane for H_2_/CH_4_ separation: Insights from molecular dynamics simulations. Int. J. Hydrog. Energy.

[125] Vinh-Thang H., Kaliaguine S. (2013). Predictive models for mixed-matrix membrane performance: A review. Chem. Rev..

[126] Cussler E.L. (1990). Membranes containing selective flakes. J. Membr. Sci..

[127] Lin H., Van Wagner E., Freeman B.D., Toy L.G., Gupta R.P. (2006). Plasticization-enhanced hydrogen purification using polymeric membranes. Science.

[128] Lin W.-H., Chung T.-S. (2001). Gas permeability, diffusivity, solubility, and aging characteristics of 6FDA-durene polyimide membranes. J. Membr. Sci..

[129] Swaidan R., Ghanem B., Pinnau I. (2015). Fine-Tuned Intrinsically Ultramicroporous Polymers Redefine the Permeability/Selectivity Upper Bounds of Membrane-Based Air and Hydrogen Separations. ACS Macro Lett..

[130] Chuah C.Y., Li W., Yang Y., Bae T.-H. (2020). Evaluation of porous adsorbents for CO_2_ capture under humid conditions: The importance of recyclability. Chem. Eng. J. Adv..

